# SETD2 transcriptional control of ATG14L/S isoforms regulates autophagosome–lysosome fusion

**DOI:** 10.1038/s41419-022-05381-9

**Published:** 2022-11-12

**Authors:** Patricia González-Rodríguez, Elizabeth Delorme-Axford, Amélie Bernard, Lily Keane, Vassilis Stratoulias, Kathleen Grabert, Pinelopi Engskog-Vlachos, Jens Füllgrabe, Daniel J. Klionsky, Bertrand Joseph

**Affiliations:** 1 Institute of Environmental Medicine, Toxicology Unit, Karolinska Institutet, 17177 Stockholm, Sweden; 2 Department of Oncology Pathology, Cancer Centrum Karolinska, Karolinska Institutet, 171 76 Stockholm, Sweden; 3 Life Sciences Institute, and the Department of Molecular, Cellular and Developmental Biology, University of Michigan, Ann Arbor, MI USA; 4 Present Address: Division of Biochemistry, Department of Molecular Medicine, Institute of Basic Medical Sciences, University of Oslo, Oslo, Norway; 5 Present Address: Department of Biological Sciences, Oakland University, Rochester, MI USA; 6 Present Address: University of Bordeaux, CNRS, Laboratoire de Biogenèse Membranaire, UMR 5200, F-33140 Villenave d’Ornon, France; 7 Present Address: Neuroscience Center, HiLIFE, University of Helsinki, Helsinki, Finland

**Keywords:** Macroautophagy, Alternative splicing

## Abstract

Macroautophagy/autophagy is an evolutionarily conserved and tightly regulated catabolic process involved in the maintenance of cellular homeostasis whose dysregulation is implicated in several pathological processes. Autophagy begins with the formation of phagophores that engulf cytoplasmic cargo and mature into double-membrane autophagosomes; the latter fuse with lysosomes/vacuoles for cargo degradation and recycling. Here, we report that yeast Set2, a histone lysine methyltransferase, and its mammalian homolog, SETD2, both act as positive transcriptional regulators of autophagy. However, whereas Set2 regulates the expression of several autophagy-related (*Atg*) genes upon nitrogen starvation, SETD2 effects in mammals were found to be more restricted. In fact, SETD2 appears to primarily regulate the differential expression of protein isoforms encoded by the *ATG14* gene. SETD2 promotes the expression of a long ATG14 isoform, ATG14L, that contains an N-terminal cysteine repeats domain, essential for the efficient fusion of the autophagosome with the lysosome, that is absent in the short ATG14 isoform, ATG14S. Accordingly, SETD2 loss of function decreases autophagic flux, as well as the turnover of aggregation-prone proteins such as mutant HTT (huntingtin) leading to increased cellular toxicity. Hence, our findings bring evidence to the emerging concept that the production of autophagy-related protein isoforms can differentially affect core autophagy machinery bringing an additional level of complexity to the regulation of this biological process in more complex organisms.

## Introduction

Macroautophagy (hereafter referred to as autophagy) is a catabolic pathway conserved from yeast to mammals, in which portions of the cytoplasm, damaged organelles, misfolded proteins, and intracellular bacteria are delivered to and degraded within the lysosomes (mammals) or vacuoles (yeast) and recycled depending on the requirement of the cells for different anabolic pathways [1]. Autophagy occurs constitutively at a basal level and can be induced by various types of cellular stress. The routine removal of cytoplasm plays a significant role in cellular homeostasis. Upon cellular stress conditions such as hypoxia, nutrient starvation, DNA damage, microbial infections, the presence of damaged or superfluous organelles or protein aggregates and even upon treatment with drugs, autophagy is upregulated and therefore considered as an adaptive mechanism [2]. Accordingly, dysfunctional autophagy is associated with a wide array of human illnesses, ranging from cancer, metabolic and heart diseases, and myopathies, to neurodegenerative disorders [3, 4].

Autophagy is comprised of a series of dynamic membrane rearrangements orchestrated by a core set of ATG (autophagy-related) proteins and other autophagy-related regulators [5]. Briefly, autophagy induction involves the activation of the class III phosphatidylinositol 3-kinase (Ptdlns3K) nucleation complex that triggers the formation of the phagophore [6, 7]. The expansion of the phagophore requires two ubiquitin-like conjugation systems, comprising the ATG12 and Atg8-family protein (MAP1LC3/LC3 and GABARAP subfamilies; we refer only to LC3 hereafter for simplicity) conjugation systems. The LC3 conjugation system involves the ATG12 conjugation system acting as an E3 ligase, which mediates the covalent attachment of phosphatidylethanolamine (PE) to Atg8 (yeast) or LC3 (mammals). As a result of membrane expansion and sealing, the autophagic cargo becomes sequestered within a double-membrane vesicle called the autophagosome. Autophagy completion involves the fusion of the mature autophagosome with a vacuole or lysosome. A set of RAB and SNARE proteins are essential for membrane tethering and fusion between autophagosomes and lysosomes [8, 9].

Epigenetic and transcriptional mechanisms are established as important regulatory events for the autophagic process (for reviews, see refs. [10**–**13]). The epigenetic and transcriptional regulation of autophagy can contribute to both short-term and long-term effects on this biological process [14**–**18]. A yeast DNA damage-responsive transcriptional repressor (Rph1) and its mammalian homolog KDM4A (lysine demethylase 4A), belong to the Jumonji C (JmjC)-domain-containing histone demethylase protein family. These proteins share the ability to demethylate a trimethyl mark on lysine 36 on histone H3 (H3K36me3); however, we previously found that the effect of Rph1 on *ATG* gene expression was not due to its histone demethylase function [19]. Rph1 and KDM4A both act as transcriptional repressors for several *ATG* genes and thereby reduce autophagic flux [20]. Whereas Rph1 and KDM4A are considered as erasers for the H3K36me3 mark, yeast Set2 (SET domain-containing 2) and its mammalian homolog counterpart SETD2/KMT3A/HYPB (SET domain containing 2, histone lysine methyltransferase) are considered to be the writers responsible for this histone post-translational modification. Set2 and SETD2 among other functions regulate epigenetic transcriptional activation, as well as play roles in the DNA damage response, and RNA alternative splicing [21, 22]. Beyond the importance of epigenetic and transcriptional regulation of the autophagic process, RNA alternative splicing is emerging as an additional contributor to the control of autophagy (for review, see ref. [23]). Therefore, mutations of SETD2 may cause genomic instability, aberrant gene transcription, RNA processing defects, and have impact on multiple biological process that have been associated with the outcome of several types of cancer such as clear cell renal cell carcinoma, glioma, lung and breast cancer [24**–**26]. However, the impact of SETD2 on autophagy and its implication on the degradation of aggregation-prone proteins such as HTT still remain elusive. Hence, we decided to decipher the role and mechanism of action for yeast Set2 and mammalian SETD2 in the control of autophagy.

## Results

### Yeast Set2 and mammalian SETD2 homologs act as positive regulators of autophagy

First, to determine the impact Set2 exerts on the autophagic process in yeast, we monitored PE conjugation to Atg8 by western blot in the presence or absence of *Set2* gene expression. Atg8 plays a role in determining the size of the autophagosome [27]. Both Atg8 and LC3 are initially synthesized as precursors that contain an additional amino acid(s) at their respective C termini; removal of this amino acid(s) by the Atg4/ATG4 protease exposes a glycine residue that is required for conjugation to PE, generating Atg8–PE and LC3–PE/LC3-II. The lipidated forms bind to both sides of the double membrane of the phagophore and control its expansion allowing the formation of the autophagosome. Atg8–PE and LC3-II are removed from the surface of the autophagosome, whereas the population trapped inside this compartment is ultimately degraded in the vacuole/lysosome. The transformation of soluble Atg8 or LC3 (LC3-I) into the lipid-bound Atg8–PE or LC3-II form is considered a hallmark of autophagy and used for monitoring the progression of this process [28].

**Fig. 1:**
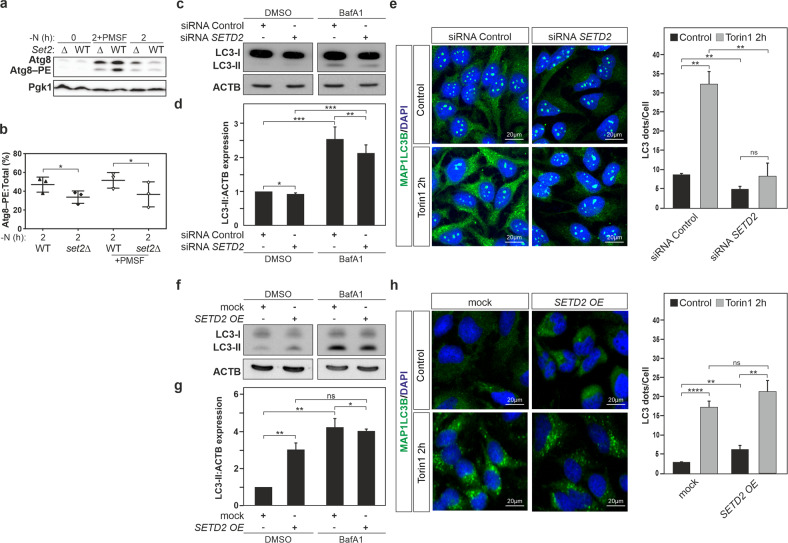
Yeast Set2 and human SETD2 act as positive regulators of autophagy. **a**, **b** Wild-type (WT; WLY176) and *set2Δ* (YAB418) yeast strains were grown in rich-medium conditions until mid-log phase. Cells were shifted to a nitrogen-starvation medium (-N) with and without PMSF that blocks vacuolar protein degradation for the indicated timepoints. **a** Protein extracts were analyzed by immunoblotting and incubated with anti-Atg8 and anti-Pgk1 (loading control) antisera. Quantification of Atg8–PE:total Atg8 ratio is shown in panel **b**. **c**, **d** Human cervical carcinoma HeLa cells were transfected with a pool of siRNAs targeting *SETD2*, or as control scramble siRNAs, for 48 h. Treatment with bafilomycin A_1_ (BafA1, 40 nM) for 4 h, which inhibits the fusion of autophagosomes with lysosomes, was used to block the autophagy flux. **c** Cells were harvested, and protein extracts analyzed by immunoblotting for LC3 and ACTB/β-actin (used as a loading control). The quantification of LC3-II (i.e., LC3–PE) levels normalized to ACTB is depicted in panel **d** and shows a significant decrease of the LC3-II:ATCB ratio in *SETD2*-deficient cells treated or not with BafA1. **e** Post-transfection with the indicated siRNA pool, cells were treated with 250 nM torin1 for 2 h or left untreated. MAP1LC3B expression was monitored by immunofluorescence analysis and LC3 puncta per cell quantified. **f**–**h** HeLa cells were transfected with an expression vector encoding SETD2, or mock-transfected with the corresponding pcDNA3.1 empty vector used as a control, for 24 h. **f** Cells were harvested, and protein extracts analyzed by immunoblotting for LC3 and ACTB (used as a loading control). The quantification of LC3-II (i.e., LC3–PE) levels normalized to ACTB is depicted in panel **g** and shows a significant increase of the LC3-II:ATCB ratio in SETD2-overexpressing HeLa cells. **h** Post-transfection with the indicated plasmids, cells were treated with 250 nM torin1 for 2 h or left untreated. MAP1LC3B expression was monitored by immunofluorescence analysis and LC3 puncta per cell quantified. **b** Bars display the mean of three independent experiments, error bars represent SD. **d**, **g** Bars display the mean of at least three independent experiments (**d**, *n* = 5; **e**, **g**, and **h** *n* = 3), error bars represent SEM; *P < 0.05; **P < 0.01; ***P < 0.001.

For the analysis of Atg8 and Atg8–PE expression levels in wild-type versus *set2∆* cells upon autophagy induction, yeast cells for these two strains were grown until mid-log phase and thereafter starved of nitrogen for 2 h. In full nutrient conditions (-N, 0 h) when autophagy is kept at a basal level, both yeast strains showed similar Atg8 expression levels (Fig. 1a). Upon nutrient limitation, both strains exhibited an increase in Atg8 and Atg8–PE expression, however, *set2*-deficient cells exhibited significantly lower induction in expression levels as well as a decrease in Atg8–PE:total Atg8 ratio (Fig. 1a, b). Because a portion of Atg8–PE will be degraded within the vacuole, a better way to assess levels of this protein is to monitor its expression during co-treatment with phenylmethanesulfonyl fluoride (PMSF) that will block vacuolar protein degradation. PMSF treatment further confirmed the reduced accumulation of Atg8–PE in *set2∆* cells. Hence, Set2 appears to work as a positive regulator of autophagy in yeast.

Thereafter, taking advantage of a gain- and loss-of-function approach, using a pool of small-interfering RNAs (siRNAs) targeting *SETD2* expression and an expression vector encoding the SETD2 protein, respectively, the impact of SETD2 on MAP1LC3B lipidation and thereby autophagy was investigated in mammalian HeLa cells. First, we confirmed the efficacy of the *SETD2/*SETD2 expression knockdown by the *SETD2* siRNA pool in HeLa cells at the messenger level by RT-qPCR analysis (Supplementary Fig. S1a) and protein level by immunoblotting analysis (Supplementary Fig. S1b). This *SETD2/*SETD2 expression knockdown was found to be associated with a very small decrease in the baseline LC3-II:ACTB ratio, as compared to cells transfected with a non-targeting control siRNA pool. However, as noted above, part of the LC3-II population is degraded in the lysosome. Therefore, to fully assess the effect of any treatment on LC3 it is necessary to also examine cells that are blocked at a late stage of autophagy, preventing the lysosomal degradation of any of the protein that was otherwise delivered to the lysosome. Treatment with bafilomycin A_1_ (BafA1) blocks the fusion of the autophagosome with the lysosome and eventually inhibits LC3-II degradation; this treatment resulted in a substantial increase in the level of LC3 in the control cells as expected (Fig. 1c, d). In SETD2-deficient cells in the presence of BafA1 there was also an increase, but the total level of LC3-II was reduced compared to the control cells. We also carried out an immunofluorescence analysis to examine the effect of SETD2 knockdown on MAP1LC3B protein expression in HeLa cells. We detected an increase in fluorescent signal for the protein and the formation of puncta representing autophagosomes upon autophagy induction following a 2-h treatment with torin1, an MTOR-dependent inducer of autophagy, that was robustly decreased in cells transfected with the *SETD2* siRNAs pool (Fig. 1e).

Second, to further assess the role for SETD2 in autophagy, the protein was overexpressed in HeLa cells; overexpression was confirmed by RT-qPCR and immunoblotting analysis (Supplementary Fig. S1c, d). Accumulation of LC3-II at the basal level was observed in HeLa cells upon SETD2 gain of function, *i.e*. overexpression, as compared to cells transfected with a control empty vector. BafA1 treatment resulted in a clear increase in LC3-II in the control cells, but there was only a modest effect in the SETD2-overexpressing cells, likely reflecting the fact that expression was already highly upregulated in the basal conditions (Fig. 1f, g). Likewise, immunofluorescence analysis for MAP1LC3B protein showed both an increased fluorescent signal for the protein as well as the formation of autophagosome-related puncta in SETD2-overexpressing cells as compared to mock-transfected cells (Fig. 1h). Collectively, these results indicate a conserved effect on autophagy, where both yeast Set2 and mammalian SETD2 act as positive regulators of this pathway.

### Yeast Set2 is required for the upregulation of expression for several *ATG* genes upon autophagy induction

**Fig. 2:**
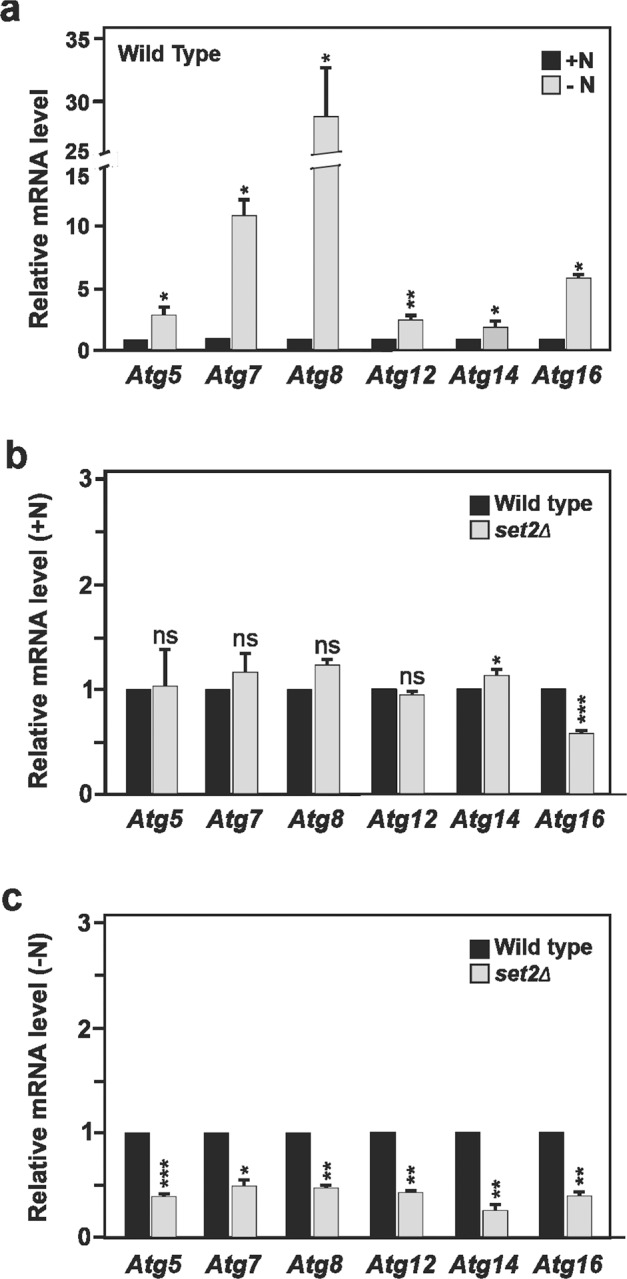
Yeast Set2 contributes to the observed upregulation of *Atg* gene expression under nutrient-deprivation conditions. **a**–**c** Wild-type (WT) and (**b**, **c**) *set2Δ* yeast strains were grown in rich medium conditions until mid-log phase. Cells were shifted to a nitrogen-starvation medium (–N) for 2 h or left in nutrient-rich conditions (+N). Thereafter, RT-qPCR analyses were performed to assess the mRNA expression levels of *Atg5*, *Atg7*, *Atg8, Atg12*, *Atg14*, and *Atg16* genes. **a** Induction of expression for all tested *Atg genes* was observed upon starvation in the WT yeast strain. **b** Under nutrient-rich conditions, *Set2* gene depletion in yeast (*set2Δ*) had, except for *Atg16*, no significant effect on the expression of the *Atg* genes tested. **c** Starved *set2Δ* yeast showed significantly lower expression for most of the tested *Atg* genes, as compared to starved WT yeast. Bars display the mean of three independent experiments (*n* = 3), error bars represent SEM; *P < 0.05; **P < 0.01; ***P < 0.001; ns: non-significant.

Considering that Set2, as with SETD2, has the ability to promote transcriptional activation and, as previously shown, it is found to contribute as a positive regulator of the autophagy process per se, we decided to investigate whether Set2 acts by regulating the expression of *Atg* genes in yeast. Upon autophagy induction in yeast such as under conditions of nitrogen starvation, the expression of *Atg* genes, including *Atg5*, *Atg7*, *Atg8*, *Atg12, Atg14*, and *Atg16*, was found to be upregulated (Fig. 2a). Under normal conditions with full nutrients, except for *Atg16*, no significant difference in gene expression was observed in *set2*-deficient cells compared to wild-type cells for any of the *Atg* genes measured (Fig. 2b). However, upon nitrogen starvation the expression level of most *Atg* genes (*Atg5*, *Atg7*, *Atg8*, *Atg12*, and *Atg16)* normally found to be upregulated upon autophagy induction (Fig. 2a) were significantly reduced in the *set2∆* cells (Fig. 2c). Thus, yeast Set2 should be considered as a positive transcriptional regulator of numerous *Atg* genes, which encode proteins exerting specific functions during several steps of the autophagic process.

### Mammalian SETD2 is a positive transcriptional regulator for the *ATG14* and *MAP1LC3B* genes

**Fig. 3:**
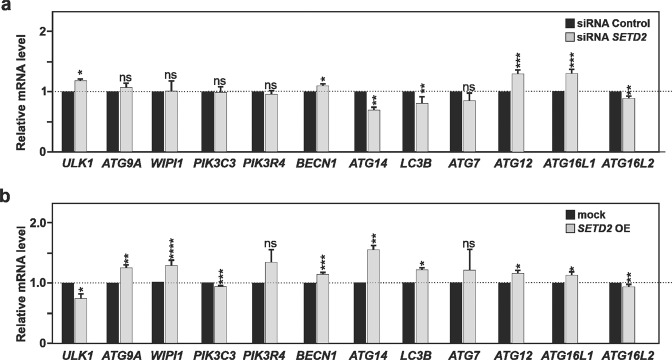
Human SETD2 acts as a positive transcriptional regulator for the *ATG14* and *MAP1LC3B* genes. **a** HeLa cells were transfected with a control non-targeting siRNA pool or *SETD2* siRNA pool for 48 h. **b** HeLa cells were transfected with an expression vector encoding SETD2, or mock transfected with the corresponding pcDNA3.1 empty vector used as control, for 24 h. **a**, **b** Cells were harvested, total RNA extracted, and the expression level for several autophagy-related genes measured by RT-qPCR analysis. These gain- and loss-of-function analyses revealed the preferential regulation of *MAP1LC3B/LC3B* and *ATG14* genes by SETD2. Bars display the mean of at least three independent experiments, error bars represent SEM; *P < 0.05; **P < 0.01; ***P < 0.001; ns: non-significant.

Considering that yeast Set2 affected the expression of several *Atg* genes under autophagy-inducing conditions and thus positively affect on the autophagy process, we decided to next examine whether a similar transcriptional effects of SETD2 may be conserved in mammalian cells. For this purpose, we focused on genes that display a clearly detectable baseline autophagy level in HeLa cells involved in different steps of the autophagy pathway, including *ATG7, ATG9, ATG14, ATG16L1, ATG16L2, BECN1, MAP1LC3B/LC3B, PI3KC3/VPS34, PIK3R4/VPS15, WIPI1*, and *ULK1*. In this mammalian setup, knockdown of *SETD2* gene expression using an siRNA pool targeting its expression, led to significant upregulation of *ATG12*, *ATG16L1*, and *ULK1*, as well as significant downregulation of *ATG14*, *ATG16L2*, and *LC3B* (Fig. 3a). In contrast, SETD2 overexpression promoted significant upregulation of *ATG9*, ATG12, *ATG14*, *ATG16L1*, *BECN1*, *LC3B*, and *WIPI1*, and significant downregulation of *ATG16L2, PIK3C3*, and *ULK1* (Fig. 3b). Whereas several autophagy-related genes appeared to be regulated by the manipulation of SETD2 expression level, only *ATG14* and *LC3B* gene expression were found to be both positively affected by SETD2 gain of function and negatively affected by its loss of function. Hence, SETD2 could be considered as a preferential transcriptional regulator of *ATG14* and *LC3B* gene expression in mammalian cells, in contrast with the results found in yeast where Set2 affected globally *Atg* expression in order to have a similar effect in the autophagy pathway.

### SETD2 promotes differential expression of ATG14 isoforms in mammals

**Fig. 4:**
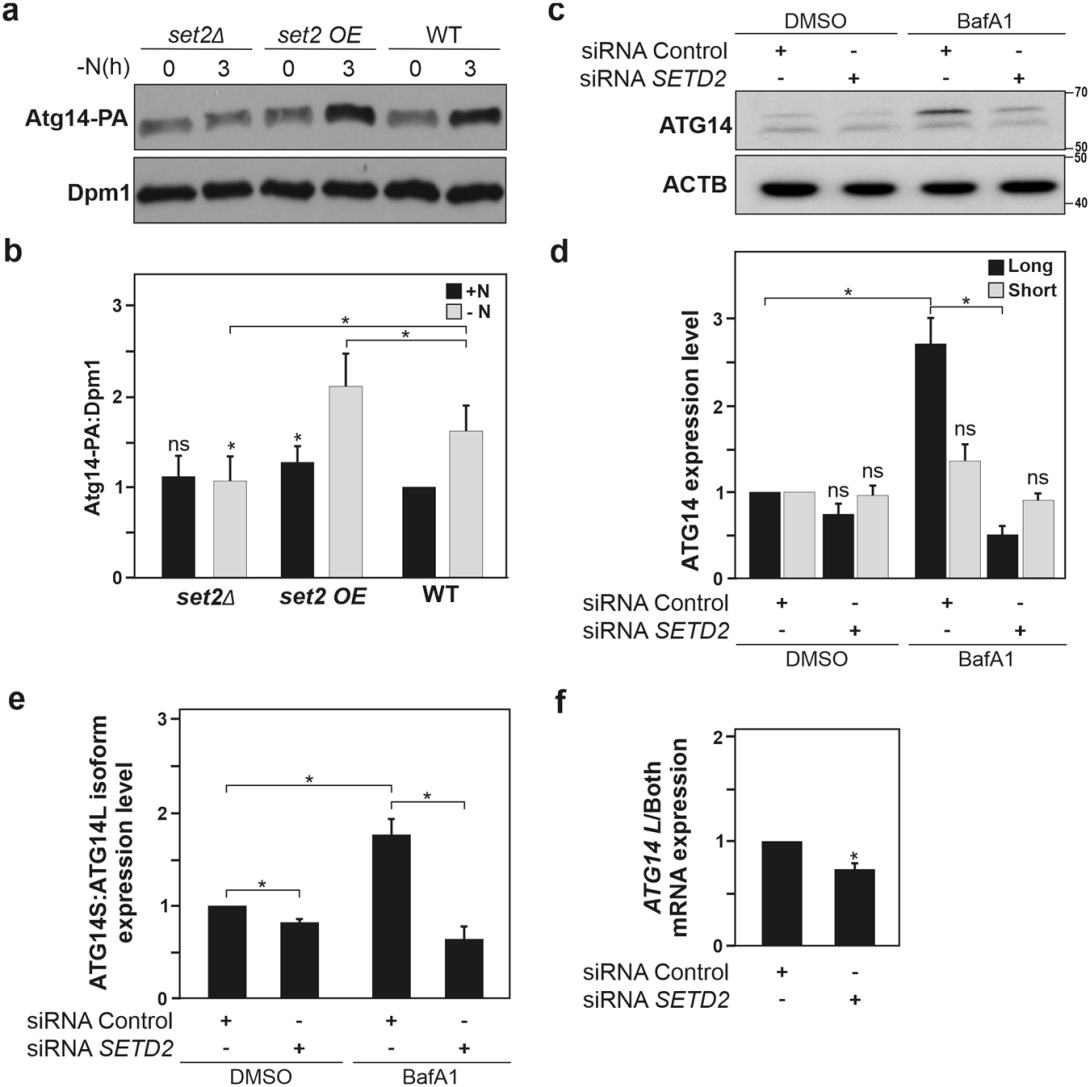
SETD2 promotes the differential expression of ATG14 protein isoforms. **a** To analyze Atg14 protein expression in WT (YAB420), *set2Δ* (YAB421) and *Set2*-overexpressing (OE; YAB422) yeast strains, a protein A (PA) tag was integrated at the chromosomal *ATG14* locus. Cells were grown in rich medium conditions (+N) until mid-log phase and then shifted to nitrogen-starvation medium (−N) for 3 h. Protein extracts were analyzed by immunoblotting and incubated with antisera that recognize PA for Atg14 detection, and anti-Dpm1 was used as a loading control. Quantification of the single Atg14-PA protein band expression levels is shown in panel **b**. **c**–**e** HeLa cells were transfected with a control non-targeting siRNA pool or *SETD2* siRNA pool for 48 h and thereafter treated with BafA1 for 4 h. **c** Cells were harvested, and protein extracts analyzed by immunoblotting for ATG14 that revealed the existence of two ATG14 immunoreactive protein bands corresponding to distinct ATG14 protein isoforms (see Fig. 6). ACTB was used as a loading control. **d**, **e** Quantification of the individual isoforms, ATG14 long isoform (ATG14L, upper band) and short isoform (ATG14S, lower band) versus ACTB was performed (**d**) or their ratio, i.e. ATG14S:ATG14L isoforms (**e**) are depicted. **f** Analysis of *ATG14L* mRNA expression versus expression of both isoforms *ATG14L* and *ATG14S* was analyzed by RT-qPCR using primer sets corresponding to the unique N-terminal domain of ATG14L or recognizing both isoform transcripts. Ct values were normalized with housekeeping genes. Bars display the mean of at least three independent experiments (**a**, **b** and **f**, *n* = 4; **c** and **e**, *n* = 5), error bars represent SEM; *P < 0.05, **P < 0.01, ***P < 0.001, ns: non-significant.

Atg14/ATG14, is an important component of the class III PtdIns3K complex known to participate in an early step of autophagy, *i.e*. vesicle nucleation [29]. However, ATG14 is also reported to be involved at later stages of this biological process with the control of membrane tethering and autophagosome-lysosome fusion to form the autolysosome [8]. Remarkably, defects in this second Atg14/ATG14-associated function could potentially contribute to the observed reduction of Atg8–PE or LC3-II in yeast and mammalian cells, respectively, as well as the apparent decrease of autophagosomes in cells (as illustrated by the accumulation of MAP1LC3B-positive puncta), in cells deficient for Set2/SETD2. Hence, the likely regulation of Atg14/ATG14 protein expression by Set2/SETD2 was further investigated in both cell systems by immunoblotting. In yeast cells, Atg14 protein expression was upregulated upon autophagy induction, using nitrogen starvation as an inducer (Fig. 4a, b). Whereas the induction of Atg14 upon nutrient limitation was not observed in the *set2∆* cells, it was further enhanced in cells overexpressing Set2.

Similarly, we sought to address whether SETD2 has an impact on ATG14 protein levels. For this purpose, immunoblot analyses of ATG14 performed in HeLa cells allowed the detection under control conditions of two distinct protein bands that upon BafA1 treatment led to the accumulation of the upper band (the band with higher molecular weight), which suggests that SETD2 could be responsible of the expression of ATG14 isoforms (Fig. 4c–e). In fact, siRNA-mediated knockdown of *SETD2* in HeLa cells selectively and significantly hindered the accumulation of the higher molecular mass ATG14 isoform (ATG14L) upon BafA1 treatment, whereas the ATG14 short (ATG14S) isoform was not affected (Fig. 4c, d). Furthermore, this analysis revealed that knockdown of SETD2 caused a small decrease in the ratio of ATG14S:ATG14L in basal conditions, but a substantial increase in the ratio of ATG14S:ATG14L in the presence of BafA_1_ (Fig. 4e). In order to further validate that ATG14 isoforms were differentially expressed at the transcript level, HeLa cells were either transfected with an siRNA pool targeting SETD2 expression or with a non-targeting siRNA pool used as a negative control. RT-qPCR analysis was performed taking advantage of primer sets designed to detect transcripts for *ATG14L* or both *ATG14* isoforms, which confirmed that upon *SETD2* knockdown, *ATG14L* mRNA expression levels were reduced in HeLa cells compared to the control cells (Fig. 4f).

**Fig. 5:**
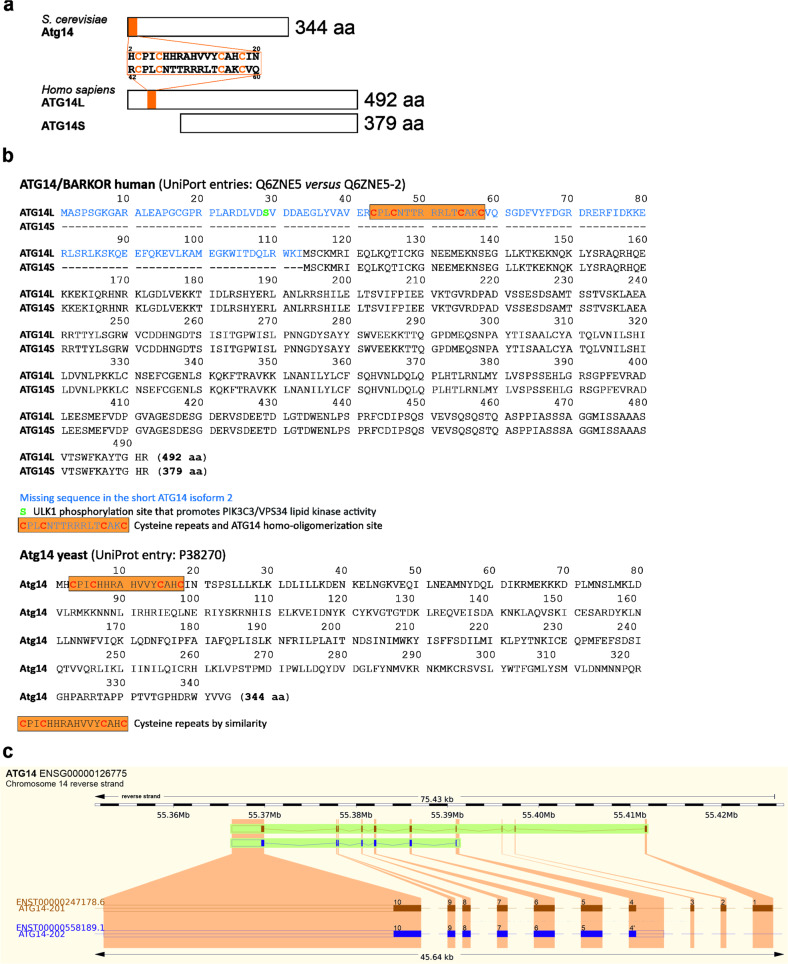
The yeast *Atg14* gene codes for one protein, whereas the human *ATG14* gene encodes two protein isoforms. Genome database searches revealed that whereas the yeast *Atg14* gene codes for one protein (344 amino acids, aa; UniProt entry: P38270), the human *ATG14* gene encodes two isoforms: a long (492 aa) and a short (379 aa) splice isoform, named ATG14L and ATG14S (UniProt entries: Q6ZNE5 and Q6ZNE5-2, respectively). **a** Schematic illustration of yeast Atg14, human ATG14L and ATG14S proteins with indication of a conserved cysteine repeats domain in the N terminus of yeast Atg14 and human ATG14L involved in ATG14 homo-oligomerization. **b** Amino acid sequences for yeast Atg14, human ATG14L and ATG14S, with indication of the missing sequence in ATG14S as compared to ATG14L, and location of the cysteine repeats domain in yeast Atg14 and human ATG14L. **c** Illustration of the structure of the exons and introns present in the *ATG14L* and *ATG14S* isoforms based on data extracted from the *Ensembl* genome browser for the human *ATG14* gene.

In fact, genome database searches revealed that, whereas the yeast *Atg14* gene encodes one protein (344 amino acids, (aa); UniProt entry: P38270), the human *ATG14* gene encodes two isoforms: a long (492 aa) and a short (379 aa) splice isoform, named ATG14L and ATG14S (UniProt entries: Q6ZNE5 and Q6ZNE5-2, respectively), which confirms the observed sizes for the two ATG14 protein bands detected in immunoblots and ATG14 expression level (Fig. 5a, b). The ATG14S isoform lacks the first 113 amino acids of the N terminus that are present in the longer ATG14L isoform. Further bioinformatic analysis uncovered that yeast Atg14 (344 aa) and the human ATG14L long isoform share a conserved cysteine repeats domain in their N-terminal region, which is absent in the human ATG14S short isoform (Fig. 5b). The *Emsembl* genome browser for vertebrate genomes revealed for the *ATG14* gene (ENSG00000126557), two transcripts (ENSG0000247178 and ENSG00000558189) corresponding to *ATG14L* and *ATG14S* which are encoded by 10 exons and 7 exons, respectively. Whereas the longest *ATG14* isoform includes 10 exons, the shortest isoform appears to skip the first three exons (1 to 3), and instead uses a different transcription start site in an alternative exon 4’ that includes intron retention (Fig. 5c and Supplementary Data File 1). It is important to note that the use of a different start exon in ATG14S means that it lacks the distinct N-terminal domain that is present in yeast Atg14 and mammalian ATG14L. In mammalian cells, this domain that contains cysteine repeats is required for the homodimerization of ATG14, and the binding of ATG14 to the soluble N-ethylmaleimide-sensitive factor attachment protein receptor (SNARE) core domain of STX17 (syntaxin 17). STX17 belongs to a set of SNARE proteins that are essential for the fusion between autophagosomes and lysosomes [8, 30]. Taken together we uncover the novel role of SETD2 as a regulator of the differential expression of ATG14 isoforms, specifically ATG14S, the expression of which was previously unknown in HeLa cells.

### SETD2 downregulation decreases autophagy flux and inhibits autophagosome-lysosome fusion

**Fig. 6:**
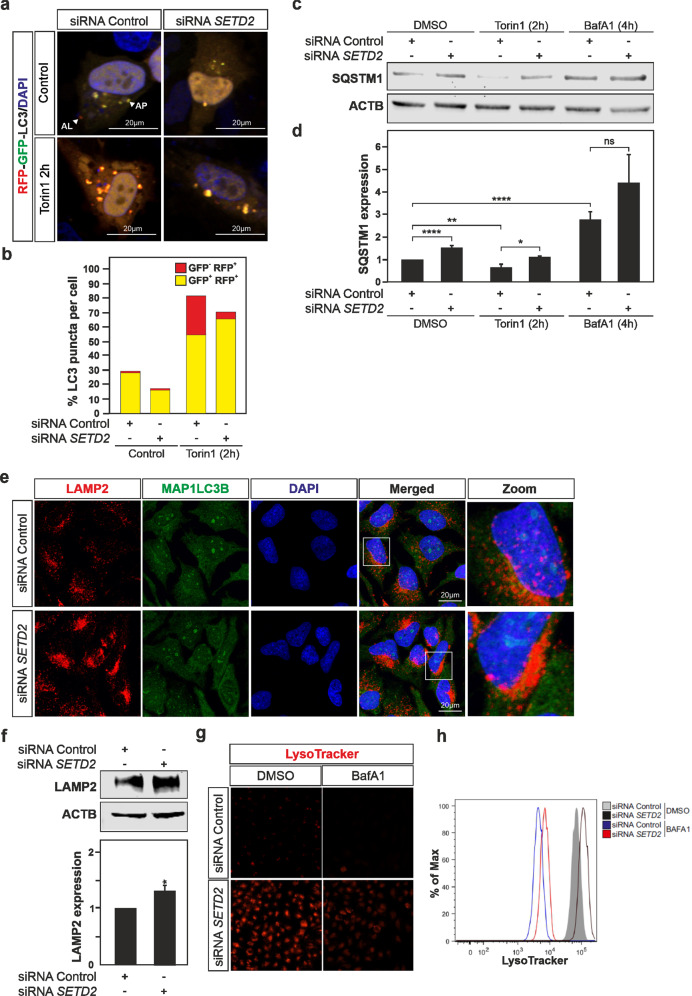
Lack of SETD2 decreases autophagic flux by inhibition of autophagosome-lysosome fusion. **a**, **b** HeLa cells were transfected with a control non-targeting siRNAs pool or *SETD2* siRNAs pool for 48 h and thereafter transfected with the mRFP-GFP-LC3 plasmid for 24 h prior to autophagy induction with torin1 for 2 h. The mRFP-GFP-LC3 tandem reporter allows distinction between autophagosomes (GFP^+^ RFP^+^; yellow puncta) and autolysosomes (GFP^−^ RFP^+^; red puncta) and was used to assess the impact of SETD2 knockdown on autophagic flux; the histogram in panel **b** represents the data of two independent experiments. **c** Immunobloting analysis of SQSTM1/p62 expression levels as a measurement of autophagic flux under baseline conditions or upon autophagy induction with torin1 for 2 h shows an increase of SQSTM1/p62 levels, corresponding to a decreased autophagy flux, in *SETD2*-deficient HeLa cells (siRNA *SETD2;* Line 2 and 4) compared to the control (siRNA Control; Lanes 1 and 3). Treatment with bafilomycin A_1_ (BafA1, 40 nM) for 4 h, which inhibits the fusion of autophagosomes with lysosomes, was used to block the autophagy flux. **d** Quantification of SQSTM1 immunoblotting. **e**, **f** HeLa cells were transfected with a control non-targeting siRNA pool or *SETD2* siRNA pool for 48 h and thereafter increased expression levels for LAMP2, a lysosomal membrane component, demonstrated by immunofluorescence imaging using phagophore bound-MAP1LC3B counterstaining (**e**) or immunoblotting using ACTB as a loading control (**f**). **g** Immunofluorescence analysis and **h** flow cytometry analysis performed on HeLa cells transfected with an siRNAs pool targeting *SETD2* expression or non-targeting siRNAs control and stained with LysoTracker^TM^ Red-fluorescent dye for labeling and tracking of acidic organelles such as lysosomes in live cells, further provided support for an accumulation of lysosomes in SETD2-deficient cells. Bars display the mean of three independent experiments (*n* = 3), error bars represent SEM; *P < 0.05, **P < 0.01, ***P < 0.001, ns: non-significant.

The above data indicate that in mammalian cells, SETD2 promotes the expression of a long ATG14 isoform, ATG14L, that contains an N-terminal cysteine repeats domain (conserved in the yeast Atg14) required for the fusion of the autophagosome with the lysosome. These observations prompted us to investigate whether SETD2 regulates autophagic flux and thus the effects on the autophagosome-lysosome fusion stage. The mRFP-GFP-LC3 tandem reporter which allows distinction between autophagosomes (GFP^+^ RFP^+^; yellow puncta) and autolysosomes (GFP^-^ RFP^+^; red puncta) was used in *SETD2*-deficient and wild-type HeLa cells, in order to assess the impact of *SETD2* knockdown on autophagic flux [31]. Torin1 treatment resulted in an increase of both yellow- and red-colored LC3 puncta in wild-type HeLa cells, indicative of autophagy induction and the occurrence of autophagic flux (Fig. 6a, b). In contrast, in *SETD2*-deficient HeLa cells the tandem reporter construct revealed that the conversion of autophagosomes to autolysosomes was reduced, consistent with a block at a late stage of the process.

To gain further support for altered autophagic flux in the *SETD2*-deficient cells, the turnover of SQSTM1/p62 (sequestosome 1) protein was investigated by immunoblotting. SQSTM1 binds directly to LC3-II and acts as an autophagic cargo receptor that recruits ubiquitinated proteins and organelles to the phagophore for degradation [32]. As a result, SQSTM1 is itself found to be degraded during the process and therefore SQSTM1 protein expression levels are used to monitor autophagy flux. Lower levels of SQSTM1 generally indicate an increase of its degradation that correlates with an increase of autophagic flux. An accumulation of SQSTM1 protein expression was observed in *SETD2*-deficient cells in basal conditions (Fig. 6c, d). RT-qPCR analysis revealed a modest yet significant increase in *SQSTM1* mRNA expression level in the *SETD2*-deficient cells that could partially contribute to the observed accumulation of SQSTM1 protein in those cells (Supplementary Fig. S2a). Following treatment with Torin1, a decrease in SQSTM1 levels were found, as expected in response to autophagy induction; however, the level of SQSTM1 remained higher in the SETD2-deficient cells suggesting a decrease of the autophagic flux in these cells relative to the control. In yeast, we previously found that Set2-deficiency was found to downregulate autophagy, so we aimed to analyze whether it has a negative impact on the autophagic flux. To monitor flux in yeast, we carried out a GFP-Atg8 processing assay. Similar to endogenous Atg8, the population of the GFP-tagged chimera present within the autophagosome is delivered to the vacuole; due to the relative protease resistance of GFP compared to Atg8, free GFP accumulates within the vacuole lumen as a marker of autophagic flux [28]. The analysis confirmed an impairment of the autophagic process in *set2Δ* cells based on the ratio of free GFP:total GFP (GFP + GFP-Atg8) as compared to wild-type cells upon nitrogen starvation, whereas Set2 overexpression did not have an appreciable effect (Supplementary Fig. 3a, b).

LAMP2 (lysosomal-associated membrane protein 2) is an important component of the lysosomal membrane and the autophagic process [33]. In LAMP2-deficient mice, autophagic vacuoles accumulate in many tissues and increased mortality between 20 and 40 days of age is observed [34]. In the absence of autophagic flux and the fusion of LC3^+^ autophagosomes with LAMP2^+^ lysosomes, accumulation of LAMP2^+^ LC3^-^ puncta are observed. Immunofluorescence analysis of both LC3 and LAMP2 in HeLa cells revealed that, upon knockdown of *SETD2* expression, the accumulation of such LAMP2-positive but LC3-negative puncta was observed, indicating an accumulation of lysosomes relative to autolysosomes in the SETD2-deficient HeLa cells (Fig. 6e). Immunoblot analysis for LAMP2 further confirmed the accumulation of this protein in *SETD2*-deficient HeLa cells (Fig. 6f). *LAMP2* mRNA expression levels analyzed by RT-qPCR were not found to be affected in the *SETD2*-deficient cells (Supplementary Fig. S2b). Immunofluorescence imaging as well as flow cytometric analysis performed on HeLa cells stained with LysoTracker^TM^ Red fluorescent dye for labeling and tracking of acidic organelles such as lysosomes in live cells, further provided support for an accumulation of lysosomes in *SETD2-*deficient cells (Fig. 6g, h). Treatment with BafA1 inhibits the vacuolar-type H^+^-translocating ATPase, thus causing a substantial increase in lysosomal pH; treatment with both BafA1 and siRNA to *SETD2* still resulted in a small increase in lysosomal accumulation. Thus, knockdown of SETD2 results in a decrease in the expression of the ATG14L isoform, affecting membrane tethering and fusion of autophagosomes with lysosomes; this leads to the relative accumulation of both compartments, and a decrease in the fusion product, autolysosomes.

### SETD2 deficiency is associated with decreased turnover of mutant HTT protein and increased cellular toxicity

**Fig. 7:**
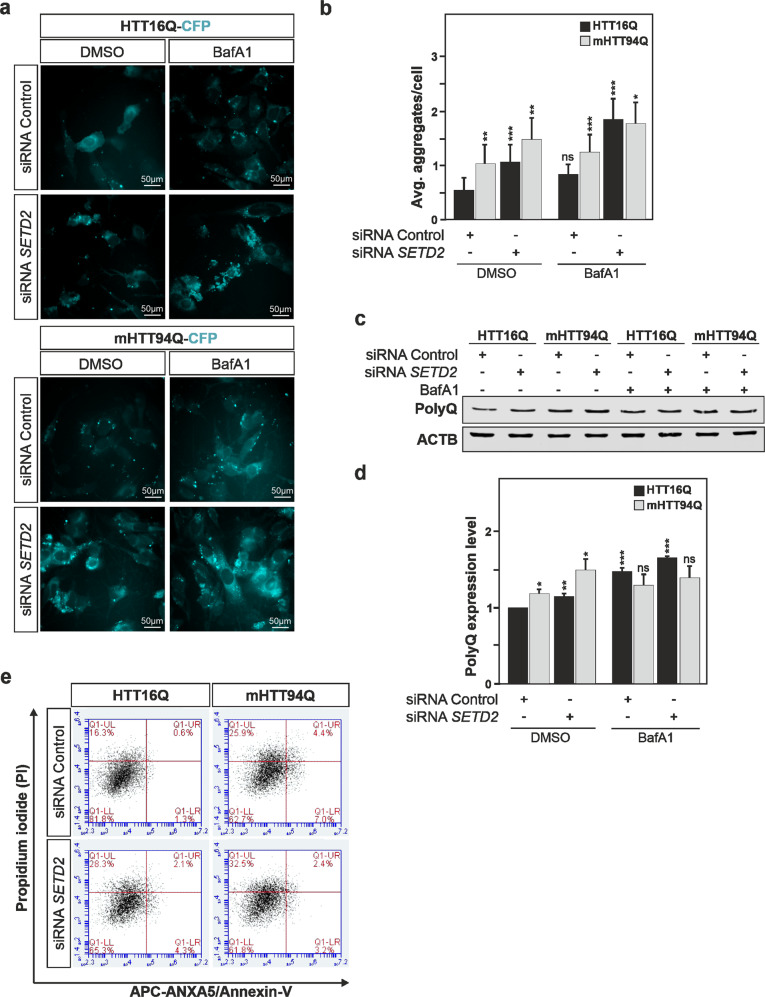
SETD2 deficiency is associated with decreased turnover of mutant HTT protein and increased cellular toxicity. **a**, **b** HeLa cells were transfected with a control non-targeting siRNAs pool or *SETD2* siRNAs pool for 48 h and thereafter transfected with the wild-type HTT16Q-CFP or disease-associated mutant mHTT94Q-CFP expression vectors for 24 h prior to protein aggregates assessment. Quantification of aggregates per cell is depicted in panel **b**. **c** Immunoblot analysis of HTT16Q and mHTT94Q expression levels in cells, under identical conditions as described above, using an antibody targeting polyQ. **d** Cell death analysis of HeLa cells transfected with wild-type HTT16Q or disease-associated mutant mHTT94Q plasmid with or without siRNA *SETD2* (a non-targeting siRNAs pool was used as a control). Propidium iodide is shown on the *Y* axis and ANXA5/annexin-V-APC on the *X* axis. Bars display the mean of at least three independent experiments (**a**–**d**, *n* = 3; **e**, *n* = 5), error bars represent SEM; *P < 0.05, **P < 0.01, ***P < 0.001.

In Huntington disease, an expansion of the polyglutamine (polyQ) tract in the N terminus of the HTT protein leads to protein aggregation. An expansion of the polyQ tract greater than 36Q is reported to cause the disease. Aggregation-prone proteins such as HTT are preferentially degraded by autophagy, but disease-inducing mutant HTT (mHTT) interrupts the process (for review, see ref. [35]). Considering that SETD2 deficiency impaired the autophagic flux and inhibited autophagosome-lysosome fusion, we decided to investigate whether SETD2 deficiency could also be associated with reduced degradation of mHTT. We examined the aggregation propensity of CFP-tagged wild-type HTT16Q and disease-inducing mHTT94Q that include a 16 and a 94 polyQ tract, respectively. Mutant HTT with a 94 polyQ tract promotes the disease in mice, whereas 16 polyQ does not [36, 37]. Immunofluorescence analysis of the CFP-tagged HTT/mHTT proteins showed that as previously reported mHTT94Q was more prone to aggregate as compared to HTT16Q in the neuronal SH-SY5Y cells (Fig. 7a, b) [36]. Immunoblot analysis for HTT/mHTT proteins using antibodies recognizing polyQ confirmed the preferential accumulation of mHTT94Q (Fig. 7c, d). Remarkably knockdown of *SETD2* in SH-SY5Y cells, led to significantly greater accumulation of the pathological mHTT94Q as well as the non-pathological HTT16Q. BafA1 treatment of the cells that blocked the autophagic flux showed a substantial increase in the accumulation of HTT16Q, whereas there was no substantial difference for mHTT94Q, suggesting that SETD2 deficiency already resulted in an essentially maximal block in turnover of the highly aggregated form of HTT.

Finally, the accumulation of protein aggregates in cells is frequently associated with increased cellular toxicity. An ANXA5/annexin V-propidium iodide (PI) flow cytometric assay was therefore performed to assess cell death. *SETD2*-deficiency was associated with increased cell death in both HTT16Q- and mHTT94Q-expressing neuronal cells (Fig. 7e).

Altogether, we uncover Set2/SETD2 as a novel positive transcriptional regulator of *Atg*/*ATG* genes, but, as a matter of complexity, SETD2 in mammals is also important for the regulation and differential expression of ATG14 isoforms, which acts as a limiting factor affecting autophagosome-lysosome fusion and thereby the autophagic flux. Although it has been demonstrated that autophagy impairment could affect the degradation and eventually the degradation of mHTT [38, 39], the transcriptional mechanism was still unknown. Here, we present for the first time SETD2 as a key player between autophagy and HTT degradation. In fact, we demonstrate that SETD2 could also contribute to the degradation of aggregation-prone proteins, and its dysfunction thereof could contribute to diseases where protein aggregation plays a pivotal part in pathology, also referred to as protein-misfolding diseases [40**–**42].

## Discussion

In a recent review, we drew attention to the fact that protein isoforms are produced by most genes encoding components of the core autophagy machinery [23]. It also appears that the production of these autophagy-related protein isoforms, can differentially affect the autophagy machinery and bring an additional level of complexity to the regulation of this biological process. Hence, the identification of a regulator(s) of the alternative splicing of pre-mRNAs originating from core autophagy-related genes is of interest.

SETD2 histone methyltransferase mediated-H3K36me3 promotes the recruitment and interaction of the splicing regulator PTBP1/HNRNP1 with MORF4L1/MRG15, a H3K36me3-binding protein in introns of transcriptionally active genes, promoting alternative splicing [43]. Decreasing SETD2 expression levels is enough to influence the inclusion of exons in genes that are otherwise alternatively spliced, ultimately leading to the generation of longer protein isoforms [44].

Here, we report that human SETD2, and its yeast homolog Set2, are positive regulators of autophagy. However, gain- and loss-of-function studies revealed that their mode of action is distinct. Indeed, while yeast Set2 acts as an inducer for transcription of several *Atg* genes, including *Atg14*, human SETD2 was able to more selectively stimulate the induction of *ATG14* gene expression and promote the generation of a long ATG14L isoform at the expense of a short ATG14S isoform. The human ATG14L isoform contains an N-terminal region with a cysteine repeats domain, conserved in yeast Atg14, but absent in the human ATG14S isoform. In a recent report, a higher molecular weight protein band was described for the ATG14 protein and proposed to result from the ULK1-dependent phosphorylation of human ATG14 at serine 29, that happens to be exclusively present in the N-terminal region of ATG14L; thus independently confirming the presence of two ATG14 protein isoforms [45].

Worth noting, approximately 95% of human multi-exon genes are subject to alternative splicing, thereby greatly expanding the coding capacity of the genome [46]. In contrast, in S. *cerevisiae* (budding yeast) only about 5% of genes contain an intron, emphasizing that the regulation of gene function *via* alternative splicing-dependent generation of protein isoforms should be considered as a regulatory mechanism significantly more developed and acquired in more complex organisms [47].

ATG14 is a bifunctional protein in autophagy regulation playing an important role in both the initiation and the completion of these biological processes, acting within the PtdIns3K complex to promote vesicle nucleation and controlling membrane tethering during autolysosome formation [8, 29]. Interestingly, the cysteine repeats domain present in yeast Atg14 and human ATG14 is reported to be involved in the homodimerization of ATG14 that is required for its binding to the SNARE protein complex, including STX17 and the mediation of autophagosome-lysosome fusion; however, no isoforms were reported previously [8]. Here we show in mammalian cells that SETD2 controls *ATG14* alternative splicing and the generation of ATG14L/S protein isoforms, which carry distinct functions that are required for the initiation and completion of autophagy offering a rather remarkable mechanism to regulate and integrate the entire biological process (Supplementary Fig. S4).

Inactivating mutations in the *SETD2* gene and impaired autophagic flux are frequent molecular and cellular features in clear cell renal cell carcinoma (ccRCC) [48]. The deficiency in SETD2 in ccRCC cells is associated with increased expression of a short ATG12 spliced isoform at the expense of the canonical long ATG12 isoform [49]. Whether the differential expression of ATG14 protein isoforms is also impaired in mutant *SETD2* ccRCC cells will require further investigation. However, it can already be noted that SETD2 expression is in both cases associated with the expression of longer protein isoforms, in agreement with the SETD2 function during alternative splicing and the reported SETD2-dependent exon(s) inclusion [42]. The current finding of the SETD2-dependent regulation of the differential expression of ATG14 isoforms, i.e. ATG14L and ATG14S, that hold distinct functions during the autophagic process may contribute to various diseases and disorders. Here, we report that *SETD2* deficiency was found to be associated with decreased turnover of mutant-HTT protein and increased cellular toxicity, offering a novel link to Huntington disease. SETD2 alterations, as well as autophagy deficiencies, have been associated with several pathophysiologies, including neurodevelopmental disorders, Luscan-Lumish syndrome and several cancer types such as renal cell carcinoma, breast cancer or leukemia [25, 26, 50–52]. Hence, our findings bring further evidence to the emerging concept that the production of autophagy-related protein isoforms can differentially affect core autophagy machinery bringing an additional level of complexity to the regulation of this biological process, as well as support to the idea that dysregulation of those regulatory mechanisms can contribute to various human diseases [23].

## Materials and methods

### Reagents

Reagents used in this study include bafilomycin A_1_ (BafA1; Santa Cruz Biotechnology, sc-201,550), Hoechst 33342 (Molecular Probes/Invitrogen, H3570), torin1 (Tocris, 4247), and PMSF (Sigma, 7626).

### Yeast strains, media, and culture

Yeast strains used in this study are listed in a supplementary Table 1 [6, 20, 53, 54]. Gene disruptions or genomic tagging were performed using a standard method [55]. Yeast cells were grown to mid-log phase in nutrient-rich medium (YPD, 1% yeast extract, 2% peptone, and 2% glucose). Autophagy was induced in yeast cells grown to mid-log phase in nutrient-rich medium and shifting to nitrogen starvation medium (SD-N, 0.17% yeast nitrogen base without amino acids, ammonium sulfate, and 2% glucose) for the indicated timepoints.

### Other methods for yeast

Protein extraction, immunoblot, and GFP-Atg8 processing were performed as previously described [20]. Antisera to Atg8 was described previously [56] and antisera to Pgk1 was a gift from Dr. Jeremy Thorner (University of California, Berkeley, USA). Monoclonal antibody to YFP (JL-8, Clontech), and a commercial antibody that reacts with PA (anti-PA, no longer available) were used as previously described [56].

### Human cell culture

Human cervical carcinoma HeLa cells (ATCC^®^, CCL-2™; RRID: CVCL_0030) and human SH-SY5Y neuroblastoma cells (ATCC^®^ CRL-2266™; RRID: CVCL_0019) were purchased from Sigma and were a gift from Dr. Thomas Perlmann (Karolinska Institutet, Stockholm, Sweden), respectively. HeLa cells and SH-SY5Y cells were grown in Minimum Essential Medium (MEM) supplemented with 1% sodium pyruvate and 1% MEM non-essential amino acids, and Dulbecco’s modified Eagle’s medium/nutrient mixture F-12 Ham (DMEM/F12), respectively. Both media were supplemented with the following: 10% fetal bovine serum (FBS), 1% L-glutamine, and 1% penicillin-streptomycin (all cell culture reagents were obtained from Gibco^®^). Cells were maintained under normal cell culture conditions in a humidified atmosphere at 37 °C degrees and 5% CO_2_. Cell lines were regularly tested for the absence of Mycoplasma using the LookOut Mycoplasma qPCR Detection Kit (Sigma Aldrich).

### Cell transfection

Cells seeded in six-well dishes were transfected with an siRNAs pool (50 nM) and 7 µL of Lipofectamine 3000 (Invitrogen) or X-tremGENE siRNA transfection reagent (Roche) on the following day. The medium was removed after 3 h of transfection and replaced with fresh medium. *SETD2* (L-012448-00), containing four different sequences that target the *SETD2* sequence, and non-targeting ON-TARGET (D-001810) SMARTpool siRNAs were purchased from Dharmacon (Supplementary Table 2). For *SETD2* overexpression experiments, HeLa cells were transfected with 2 µg of a *SETD2*-containing plasmid (gift from Dr. Xiaobing Shi, MD Anderson Cancer Center, Houston, Texas, USA) or the corresponding pcDNA3.1 empty vector used as mock using 6 µl XtremeGene HP Reagent (Roche).

### Immunoblotting

Cells were harvested at 24- or 48-h post-transfection using a cell scraper and lysed directly in Laemmli loading buffer (62.5 mM Tris-HCl, pH 6.8, 2% SDS, 10% glycerol, 5% β-mercaptoethanol, and 0.02% Bromophenol Blue). For immunoblot analysis, protein extracts were sonicated (Bioruptor^®^ Pico, Diagenode), and boiled at 96 °C for 6 min. Proteins were resolved by 8% or 15% SDS–polyacrylamide gel electrophoresis and blotted onto 0.2-µm or 0.45-µm pore-size nitrocellulose membranes in a wet transfer system (Bio-Rad). Membranes were blocked for 1 h with 5% milk in PBS-0.1% Tween 20 (PBS-T) or Animal Free Blocker (Vector). For protein detection, membranes were incubated overnight at 4 °C with the corresponding primary antibody (Supplementary Table 3) [57]. The day after, membranes were washed three times with PBS-T and incubated with the appropriate secondary antibody, RDye^®^ 680RD goat anti-rabbit or Rdye^®^ 800CW goat anti-mouse IgG (1:5000; LI-COR Bioscience) for 1 h at room temperature. Visualization was obtained using an Odyssey CLx infrared imaging system (LI-COR Bioscience). Analysis was made using anti-ACTB/β-actin as housekeeping protein for standardization of protein loading, and densitometry was done using the ImageJ software normalizing the protein of interest to the loading control, both in the same linear range of acquisition. Full length immunoblot membranes are presented in Supplementary Data File 2.

### Tandem cell fluorescence reporter flux assay

Cells were grown on coverslips in six-well dishes and after 24 h were transfected using 2 µg of the mRFP-GFP-LC3 plasmid and 6 µl of XtremeGene HP Reagent per well. The medium was changed 3 h after transfection. On the following day, cells were transfected with siRNA as previously described. The next day, cells were treated with DMSO or torin1 (250 nM) for 24 h. Cells were fixed in 4% paraformaldehyde for 7 min at room temperature, followed by nuclei staining using Hoechst. Autophagy flux was determined counting the number of LC3-positive cells. The mRFP-GFP-LC3 plasmid was a kind gift of Dr. Tamotsu Yoshimori (National Institute of Genetics, Mishima, Japan) [31].

### HTT protein aggregates assay

Cells were grown on coverslips in six-well dishes, and after 24 h cells were transfected with siRNA as previously described using Lipofectamine RNAiMAX (Invitrogen). The following day, cells were transfected using 2 µg of the HTT16Q-CFP (wild-type) or the mHTT94Q-CFP (disease-associated mutant) plasmid and 6 µl of Lipofectamine PLUS Reagent (Roche) per well. The next day, cells were fixed in 4% paraformaldehyde for 7 min at room temperature, followed by nuclei staining using Hoechst. HTT versus mHTT aggregates formation was determined counting the number of CFP-positive aggregates per cell. The HTT16Q-CFP and the mHTT94Q-CFP plasmid were kind gifts of Nico Dantuma (Karolinska Institutet, Stockholm, Sweden).

### RNA isolation and RT-qPCR

Total RNA was extracted using the RNeasy kit (Qiagen) and performing an additional DNase digestion step with the RNase-free DNase set (Qiagen). One µg of RNA was reverse-transcribed into cDNA using superscript III or IV Reverse Transcriptase (Invitrogen). qPCR reactions were carried out on an ABI 7500 and results were normalized according to *GAPDH* housekeeping gene quantification. Specific primers were predesigned oligos (used 4 nM/well, from primer stock of 100 µM) purchased from Sigma (KiCqStart). qPCR statistical analysis was done using R. Primer sequences used in the study are shown in Supplementary Table 4. Yeast RT-qPCR, including primers used, was performed as previously described [56].

### Immunofluorescence

For confocal microscopy analysis, HeLa cells were grown on coverslips. After 24 h, cells were harvested and fixed either with 4% paraformaldehyde at room temperature or using cold methanol. Next, samples were blocked in Permeabilization/Blocking buffer (10 mM HEPES, pH 7.2, 3% bovine serum albumin, 0.3% Triton X-100 diluted in PBS) at room temperature for 1 h and incubated with the indicated primary (4°C, overnight) and secondary Alexa Fluor® 488 goat anti-rabbit (1:1000; Molecular Probes/Invitrogen; room temperature, 1 h) antibodies. Antibodies are listed in Supplementary Table 3. Nuclei were counterstained with DAPI or Hoechst 33342 and were mounted with Vectashield (Vector Laboratories). Analysis was performed with Zeiss LSM700 confocal laser scanning microscopy. LC3 puncta per cells was assayed using the ImageJ software.

### Cell death

Human SH-SY5Y neuroblastoma cells were subsequently transfected with an siRNA control pool or one targeting *SETD2* and thereafter with wild-type HTT16Q or disease-associated mutant mHTT94Q. Cell death was assessed using the APC Annexin V apoptosis detection kit with PI from BioLegend (640932). Briefly, the supernatant, potentially containing dead cells, was collected, and adherent cells were removed using trypsinization. Cells were then stained, by gentle vortexing, in ANXA5/annexin V binding buffer containing ANXA5 conjugated to APC and propidium iodide for 15 min at room temperature in the dark. Samples were immediately analyzed with the BD LSR2 cell analyzer (BD Biosciences, v10).

### Statistical analysis

Statistical analysis was performed using GraphPad Prism (GraphPad Software, Version 6.0), the threshold for statistical significance was considered when the *P* value was less than 0.05; *P < 0.05, **P < 0.01, ***P < 0.001. If two conditions were to be compared, two-tailed unpaired Student *t* test was used. Two-way ANOVA was used for multiple comparisons. No samples were excluded from the analyses performed. The number of experimental repeats is described in the relevant figure legends. The investigators were not blinded to allocation during experiments and outcome assessment.

### Reporting summary

Further information on research design is available in the Nature Research Reporting Summary linked to this article.

## Supplementary information

Supp Figures S1 to S4 and Table S1 to S4

Supplementary data file 1

Uncuted Immunoblots

Reporting summary

Authors' agreement

## Data Availability

Immunoblot membranes are presented in Supplementary Data File 2. All other data are available from the corresponding author upon reasonable request.

## References

[1] Xie Z, Klionsky DJ (2007). Autophagosome formation: core machinery and adaptations. Nat Cell Biol.

[2] Mizushima N, Levine B, Cuervo AM, Klionsky DJ (2008). Autophagy fights disease through cellular self-digestion. Nature.

[3] Levine B, Kroemer G (2008). Autophagy in the pathogenesis of disease. Cell.

[4] Yoshii SR, Mizushima N (2015). Autophagy machinery in the context of mammalian mitophagy. Biochim Biophys Acta.

[5] Mizushima N, Yoshimori T, Ohsumi Y (2011). The role of Atg proteins in autophagosome formation. Annu Rev Cell Dev Biol.

[6] He C, Baba M, Klionsky DJ (2009). Double duty of Atg9 self-association in autophagosome biogenesis. Autophagy.

[7] Feng Y, He D, Yao Z, Klionsky DJ (2014). The machinery of macroautophagy. Cell Res.

[8] Diao J, Liu R, Rong Y, Zhao M, Zhang J, Lai Y (2015). ATG14 promotes membrane tethering and fusion of autophagosomes to endolysosomes. Nature.

[9] Jiang W, Chen X, Ji C, Zhang W, Song J, Li J, et al. Key regulators of autophagosome closure. Cells. 2021;10:2814. 10.3390/cells10112814 PMC8616111 34831036

[10] Füllgrabe J, Klionsky DJ, Joseph B (2014). The return of the nucleus: transcriptional and epigenetic control of autophagy. Nat Rev Mol Cell Biol.

[11] Di Malta C, Cinque L, Settembre C (2019). Transcriptional regulation of autophagy: mechanisms and diseases. Front Cell Dev Biol.

[12] Shi Y, Shen H-M, Gopalakrishnan V, Gordon N (2021). Epigenetic regulation of autophagy beyond the cytoplasm: a review. Front Cell Dev Biol.

[13] Baek SH, Kim KIL (2017). Epigenetic control of autophagy: nuclear events gain more attention. Mol Cell.

[14] Füllgrabe J, Lynch-Day MA, Heldring N, Li W, Struijk RB, Ma Q (2013). The histone H4 lysine 16 acetyltransferase hMOF regulates the outcome of autophagy. Nature.

[15] Shin H-JR, Kim H, Oh S, Lee J-G, Kee M, Ko H-J (2016). AMPK-SKP2-CARM1 signalling cascade in transcriptional regulation of autophagy. Nature.

[16] Artal-Martinez de Narvajas A, Gomez TS, Zhang J-S, Mann AO, Taoda Y, Gorman JA (2013). Epigenetic regulation of autophagy by the methyltransferase G9a. Mol Cell Biol.

[17] González-Rodríguez P, Cheray M, Füllgrabe J, Salli M, Engskog-Vlachos P, Keane L (2021). The DNA methyltransferase DNMT3A contributes to autophagy long-term memory. Autophagy.

[18] González-Rodríguez P, Cheray M, Keane L, Engskog-Vlachos P, Joseph B. ULK3-dependent activation of GLI1 promotes DNMT3A expression upon autophagy induction. Autophagy. 2022;1–12. 10.1080/15548627.2022.2039993. Epub ahead of print. 10.1080/15548627.2022.2039993 PMC9673947 35226587

[19] Edmunds JW, Mahadevan LC, Clayton AL (2008). Dynamic histone H3 methylation during gene induction: HYPB/Setd2 mediates all H3K36 trimethylation. EMBO J.

[20] Bernard A, Jin M, González-Rodríguez P, Füllgrabe J, Delorme-Axford E, Backues SK (2015). Rph1/KDM4 mediates nutrient-limitation signaling that leads to the transcriptional induction of autophagy. Curr Biol.

[21] Li J, Duns G, Westers H, Sijmons R, van den Berg A, Kok K (2016). SETD2: an epigenetic modifier with tumor suppressor functionality. Oncotarget.

[22] Lam UTF, Chen ES (2022). Molecular mechanisms in governing genomic stability and tumor suppression by the SETD2 H3K36 methyltransferase. Int J Biochem Cell Biol.

[23] González-Rodríguez P, Klionsky DJ, Joseph B (2022). Autophagy regulation by RNA alternative splicing and implications in human diseases. Nat Commun.

[24] Fontebasso AM, Schwartzentruber J, Khuong-Quang DA, Liu XY, Sturm D, Korshunov A (2013). Mutations in SETD2 and genes affecting histone H3K36 methylation target hemispheric high-grade gliomas. Acta Neuropathol.

[25] Morris MR, Latif F (2017). The epigenetic landscape of renal cancer. Nat Rev Nephrol.

[26] Zhu X, He F, Zeng H, Ling S, Chen A, Wang Y (2014). Identification of functional cooperative mutations of SETD2 in human acute leukemia. Nat Genet.

[27] Xie Z, Nair U, Klionsky DJ (2008). Atg8 controls phagophore expansion during autophagosome formation. Mol Biol Cell.

[28] Klionsky DJ, Abdel-Aziz AK, Abdelfatah S, Abdellatif M, Abdoli A, Abel S (2021). Guidelines for the use and interpretation of assays for monitoring autophagy (4th edition)1. Autophagy.

[29] Sun Q, Fan W, Chen K, Ding X, Chen S, Zhong Q (2008). Identification of Barkor as a mammalian autophagy-specific factor for Beclin 1 and class III phosphatidylinositol 3-kinase. Proc Natl Acad Sci USA.

[30] Nair U, Jotwani A, Geng J, Gammoh N, Richerson D, Yen W-L (2011). SNARE proteins are required for macroautophagy. Cell.

[31] Kimura S, Noda T, Yoshimori T. Dissection of the autophagosome maturation process by a novel reporter protein, tandem fluorescent-tagged LC3. Autophagy. 2007;3:452–60. 10.4161/auto.4451 17534139

[32] Pankiv S, Clausen TH, Lamark T, Brech A, Bruun J-A, Outzen H (2007). p62/SQSTM1 binds directly to Atg8/LC3 to facilitate degradation of ubiquitinated protein aggregates by autophagy. J Biol Chem.

[33] Eskelinen E-L, Illert AL, Tanaka Y, Schwarzmann G, Blanz J, Von Figura K (2002). Role of LAMP-2 in lysosome biogenesis and autophagy. Mol Biol Cell.

[34] Tanaka Y, Guhde G, Suter A, Eskelinen EL, Hartmann D, Lüllmann-Rauch R (2000). Accumulation of autophagic vacuoles and cardiomyopathy in LAMP-2-deficient mice. Nature.

[35] Martin DDO, Ladha S, Ehrnhoefer DE, Hayden MR (2015). Autophagy in Huntington disease and huntingtin in autophagy. Trends Neurosci.

[36] Li L, Liu H, Dong P, Li D, Legant WR, Grimm JB (2016). Real-time imaging of Huntingtin aggregates diverting target search and gene transcription. ELife.

[37] Pitzer M, Lueras J, Warden A, Weber S, McBride J (2015). Viral vector mediated expression of mutant huntingtin in the dorsal raphe produces disease-related neuropathology but not depressive-like behaviors in wildtype mice. Brain Res.

[38] Iwata A, Christianson JC, Bucci M, Ellerby LM, Nukina N, Forno LS (2005). Increased susceptibility of cytoplasmic over nuclear polyglutamine aggregates to autophagic degradation. Proc Natl Acad Sci USA.

[39] Wold MS, Lim J, Lachance V, Deng Z, Yue Z (2016). ULK1-mediated phosphorylation of ATG14 promotes autophagy and is impaired in Huntington’s disease models. Mol Neurodegener.

[40] Chaudhuri TK, Paul S (2006). Protein-misfolding diseases and chaperone-based therapeutic approaches. FEBS J.

[41] Menzies FM, Moreau K, Rubinsztein DC (2011). Protein misfolding disorders and macroautophagy. Curr Opin Cell Biol.

[42] Ciechanover A, Kwon YT (2015). Degradation of misfolded proteins in neurodegenerative diseases: therapeutic targets and strategies. Exp Mol Med.

[43] Iwamori N, Tominaga K, Sato T, Riehle K, Iwamori T, Ohkawa Y (2016). MRG15 is required for pre-mRNA splicing and spermatogenesis. Proc Natl Acad Sci USA.

[44] Luco RF, Pan Q, Tominaga K, Blencowe BJ, Pereira-Smith OM, Misteli T (2010). Regulation of alternative splicing by histone modifications. Science.

[45] Park J-M, Jung CH, Seo M, Otto NM, Grunwald D, Kim KH (2016). The ULK1 complex mediates MTORC1 signaling to the autophagy initiation machinery via binding and phosphorylating ATG14. Autophagy.

[46] Wang ET, Sandberg R, Luo S, Khrebtukova I, Zhang L, Mayr C (2008). Alternative isoform regulation in human tissue transcriptomes. Nature.

[47] Spingola M, Grate L, Haussler D, Ares M (1999). Genome-wide bioinformatic and molecular analysis of introns in Saccharomyces cerevisiae. RNA.

[48] Cancer Genome Atlas Research Network (2013). Comprehensive molecular characterization of clear cell renal cell carcinoma. Nature.

[49] González-Rodríguez P, Engskog-Vlachos P, Zhang H, Murgoci A-N, Zerdes I, Joseph B (2020). SETD2 mutation in renal clear cell carcinoma suppress autophagy via regulation of ATG12. Cell Death Dis.

[50] Passani LA, Bedford MT, Faber PW, McGinnis KM, Sharp AH, Gusella JF (2000). Huntingtin’s WW domain partners in Huntington’s disease post-mortem brain fulfill genetic criteria for direct involvement in Huntington’s disease pathogenesis. Hum Mol Genet.

[51] Dalgliesh GL, Furge K, Greenman C, Chen L, Bignell G, Butler A (2010). Systematic sequencing of renal carcinoma reveals inactivation of histone modifying genes. Nature.

[52] Lumish HS, Wynn J, Devinsky O, Chung WK (2015). Brief report: SETD2 mutation in a child with autism, intellectual disabilities and epilepsy. J Autism Dev Disord.

[53] Mao K, Chew LH, Inoue-Aono Y, Cheong H, Nair U, Popelka H (2013). Atg29 phosphorylation regulates coordination of the Atg17-Atg31-Atg29 complex with the Atg11 scaffold during autophagy initiation. Proc Natl Acad Sci USA.

[54] Robinson JS, Klionsky DJ, Banta LM, Emr SD (1988). Protein sorting in *Saccharomyces cerevisiae*: isolation of mutants defective in the delivery and processing of multiple vacuolar hydrolases. Mol Cell Biol.

[55] Longtine MS, McKenzie A, Demarini DJ, Shah NG, Wach A, Brachat A (1998). Additional modules for versatile and economical PCR-based gene deletion and modification in *Saccharomyces cerevisiae*. Yeast.

[56] Lahiri V, Metur SP, Hu Z, Song X, Mari M, Hawkins WD, et al. Post-transcriptional regulation of ATG1 is a critical node that modulates autophagy during distinct nutrient stresses. Autophagy. 2021;18:1694–714. 10.1080/15548627.2021.1997305 PMC9298455 34836487

[57] Huang WP, Scott SV, Kim J, Klionsky DJ (2000). The itinerary of a vesicle component, Aut7p/Cvt5p, terminates in the yeast vacuole via the autophagy/Cvt pathways. J Biol Chem.

