# Supplementary material for: SETD2 transcriptional control of ATG14L/S isoforms regulates autophagosome–lysosome fusion
Source: Cell Death Dis. 2022 Nov 12;13(11):953. doi: 10.1038/s41419-022-05381-9 (PMC9653477; doi:10.1038/s41419-022-05381-9)
Supplement: Supplementary file 1 — Supp Figures S1 to S4 and Table S1 to S4 [file 41419_2022_5381_MOESM1_ESM.docx]

**
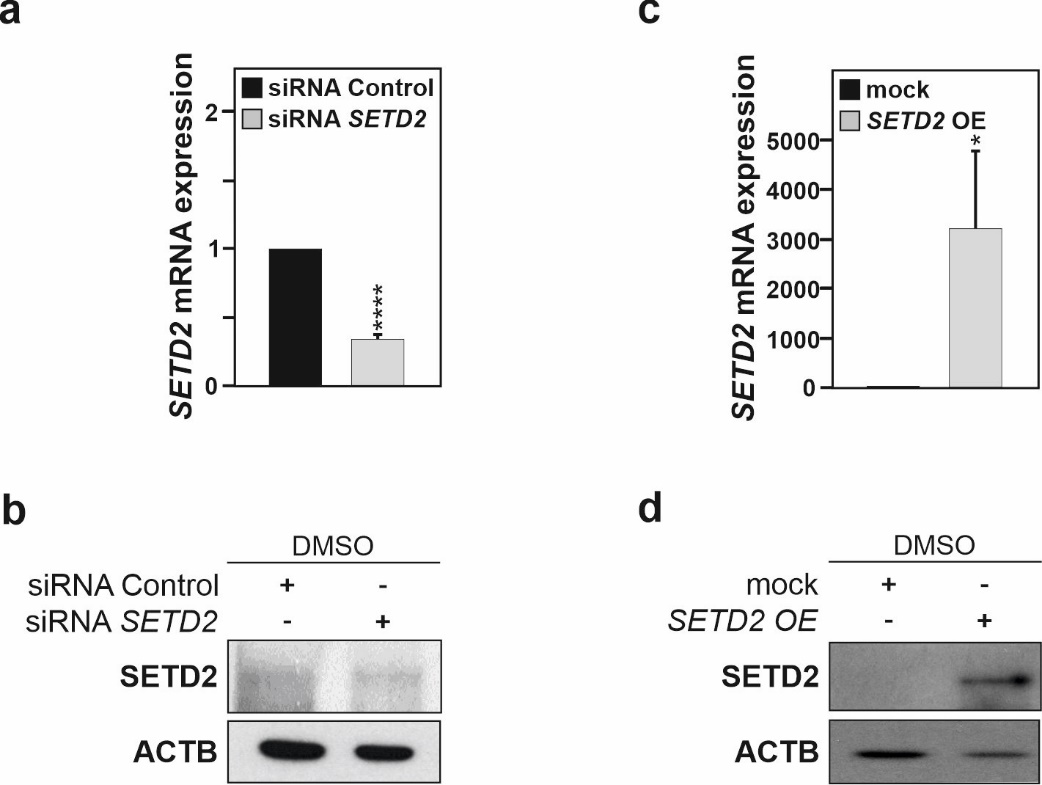
**

**Supplementary figure S1 | Validation of *SETD2* knockdown or overexpression in HeLa cells.**

(**a** and **b**) HeLa cells were transfected with a control non-targeting siRNAs pool or *SETD2* siRNAs pool for 48 h. (**a**) RT-qPCR analysis for *SETD2* mRNA expression level confirmed efficient knockdown in siRNA *SETD2*-transfected HeLa cells as compared with the siRNA control-transfected ones. (**b**) Immunoblot analysis for SETD2 and ACTB/β-actin (used as a loading control) show a decreased expression level for SETD2 in HeLa cells with the *SETD2* siRNAs pool. (**c** and **d**) HeLa cells were transfected with an expression vector encoding SETD2, or mock transfected with the corresponding pcDNA3.1 empty vector used as a control, for 24 h. (**c**) RT-qPCR analysis for *SETD2* mRNA expression level confirmed efficient upregulation of *SETD2* expression in HeLa cells transfected with a SETD2 expression vector as compared with the mock-transfected ones. (**d**) Immunoblot analysis for SETD2 and ACTB/β-actin show an increased expression level for SETD2 in SETD2-overexpressing HeLa cells.

Bars display the mean of 8 (a) and 4 (c) independent experiments, error bars represent SEM; **p*<0.05; ***p < 0.001.

**
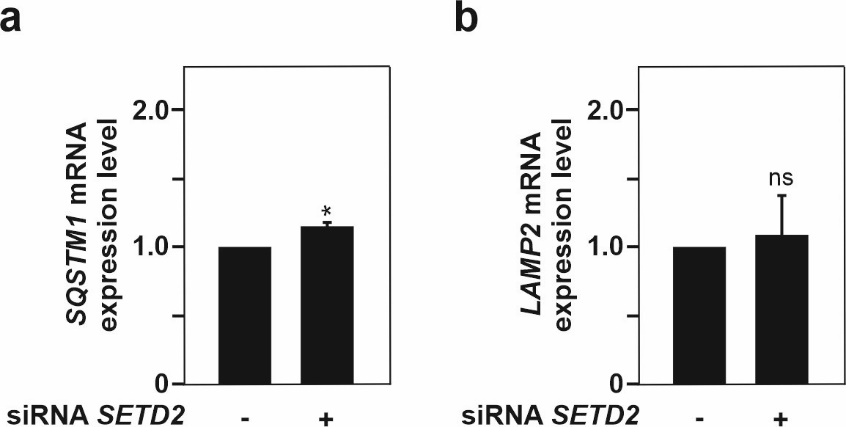
**

**Supplementary figure S2 | SQSTM1 and LAMP2 mRNA expression in HeLa cells upon *SETD2* knockdown.**

(**a** and **b**) HeLa cells were transfected with a control non-targeting siRNAs pool or *SETD2* siRNAs pool for 48 h. (**a**) RT-qPCR analysis for *SQSTM1* mRNA expression level indicated a modest, yet significant increase in siRNA *SETD2*-transfected HeLa cells as compared with the siRNA control-transfected ones. (**b**) RT-qPCR analysis for *LAMP2* mRNA expression level did not show any significant difference in expression levels between siRNA *SETD2*-transfected HeLa cells and siRNA control-transfected ones.

Bars display the mean of three independent experiments, error bars represent SEM; **p*<0.05.

**
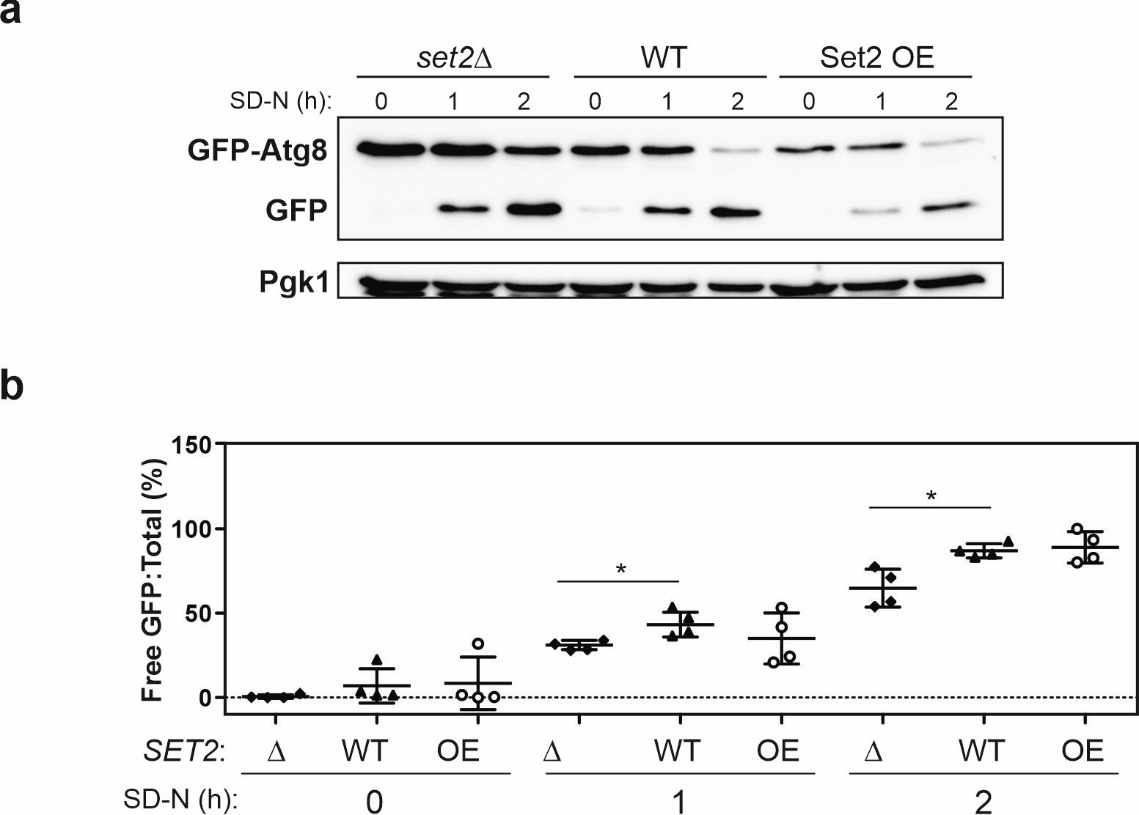
**

**Supplementary figure S3 | Lack of Set2 decreases autophagic flux in yeast.**

(**a** and **b**) WT (WLT176), *set2Δ* (YAB418) and *Set2* OE (YAB419) yeast strains were transformed with a centromeric plasmid encoding GFP-Atg8 under the control of the endogenous *ATG8* promoter. Cells were grown overnight to mid-log phase in rich selective medium and shifted to nitrogen-starvation conditions (-N) for the indicated period. Protein extracts were analyzed by immunoblotting and incubated with anti-GFP and anti-Pgk1 (loading control) antisera. Quantification of free GFP:total (GFP-Atg8 + free GFP) ratio from 4 independent experiments is depicted in panel B.

**
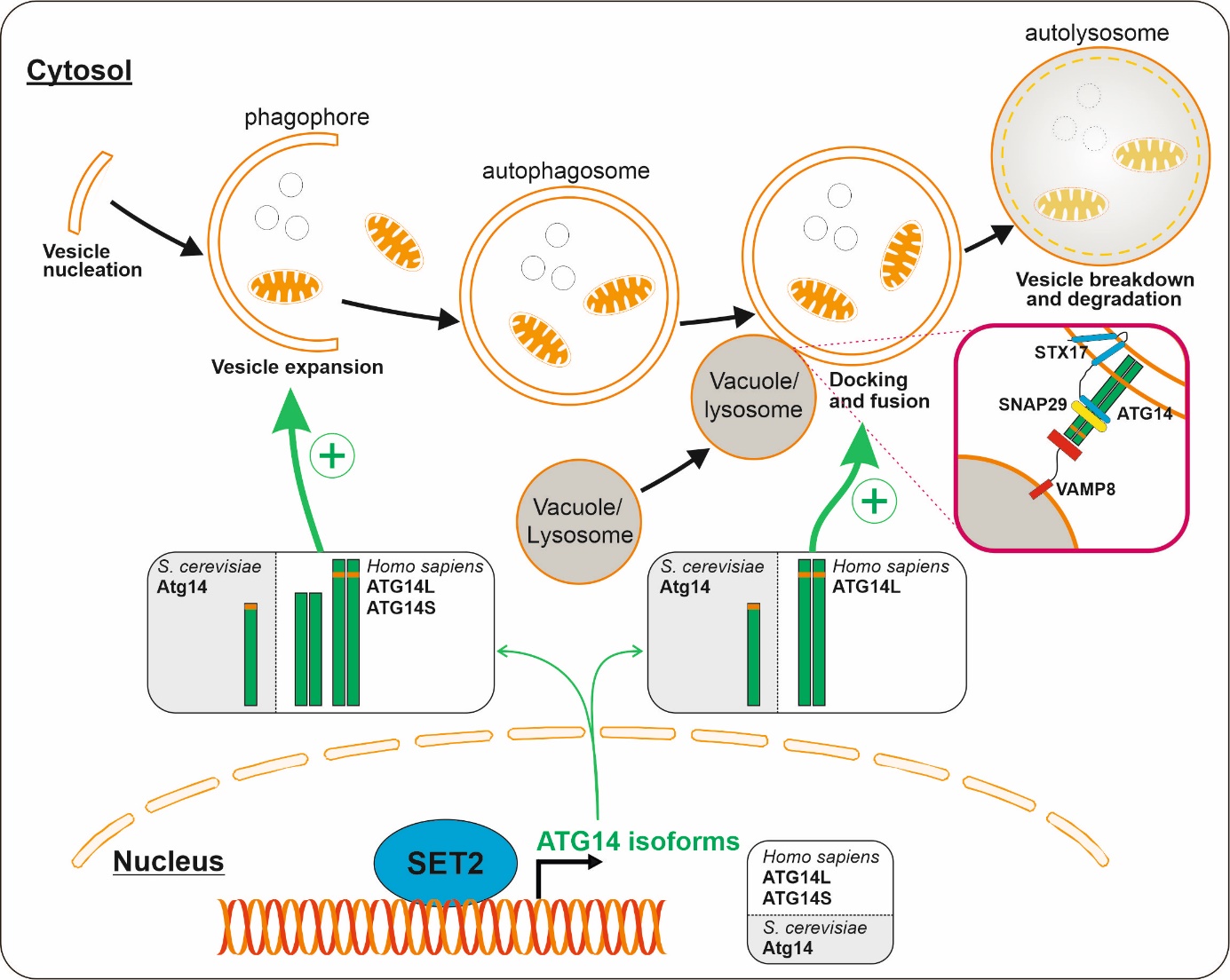
**

**Supplementary figure S4 | Schematic illustration of the proposed mechanism.** Illustration of the mechanism evolutionarily conserved from yeast to mammals. Set2/SETD2 promotes the expression of Atg14/ATG14 in both systems. In mammals due to the complexity of the system over the course of evolution, SETD2 promotes the differential expression of ATG14 isoforms. As shown, the ATG14 long isoform (ATG14L) is involved in both vesicle expansion and docking and fusion of the autophagosome with the lysosome by allowing the interaction of STX17-SNAP29 with VAMP8. in contrast, the short isoform (ATG14S) is only involved in vesicle expansion as it does not contain the cysteine-rich domain (in orange) required for ATG14 homo-oligomerization.

**SUPPLEMENTARY TABLES**

**Table 1 | Yeast strains used in this study** ^6,20,61,62^

| **Name** | **Genotype** | **Reference** |
| --- | --- | --- |
| BY4742 | MATα *his3Δ1 leu2Δ0 ura3Δ0* | ResGen/Invitrogen |
| SEY6210 | MATα *leu2-3,112 ura3-52 his3-Δ200 trp1-Δ901 suc2-Δ9 lys2-801; GAL* | Ref 54 |
| WLY176 | SEY6210 *pho13∆ pho8::pho8∆60* | Ref 53 |
| YAB318 | *set2Δ::His* *pho13Δ::Kan pho8Δ60* | This study |
| YAB348 | YTS158, *Atg14-PA::HIS5* | This study |
| YAB349 | YAB300, *Atg14-PA::HIS5* | Ref 20 |
| YAB420 | SEY6210 *Atg14-PA::HIS5 pho13Δ pho8Δ60* | This study |
| YAB421 | SEY6210 *Atg14-PA::HIS5 set2∆::TRP1 pho13Δ pho8Δ60* | This study |
| YAB422 | SEY6210 *Atg14-PA::HIS5 ZEO1p::Kan-Set2 pho13Δ pho8Δ60* | This study |
| YTS158 | BY4742, *pho13∆::KanMX6 pho8::pho8∆60* | Ref 6 |

**Table 2 | Antibodies used in this study**

|  | **Research Resource Identifier** | **Application** | **Sources** |
| --- | --- | --- | --- |
| **ACTB/β-actin** (mouse mAb) | RRID:AB_262137 | IB | Sigma Aldrich (A-3853) |
| **Atg8** (yeast) |  | IB | Ref 56 |
| **ATG14** (rabbit pAb) | RRID:AB_10695397 | IB | Cell Signaling Technology (#5504) |
| **Dpm1** (mouse mAb) | RRID:AB_2536204 | IB | Molecular Probes/Fisher (A6429) |
| **LAMP2** (mouse mAb) | RRID:AB_470709 | IB, IF | Abcam (ab25631) |
| **LC3B** (rabbit pAb) | RRID:AB_796155 | IB | Sigma Aldrich (L7543) |
| **SETD2** (rabbit pAb) | RRID:AB_2811237 | IB | Genetex (GTX127905) |
| **Protein A (PA)** (rabbit pAb) | RRID:AB_2315781 | IB | Jackson Immunoresearch (323-005-024)^a^ |
| **PolyQ** (mouse mAb) | RRID:AB_2920696 | IB | Millipore (MABN2427) |
| **SQSTM1/p62** (mouse mAb) | RRID:AB_945626 | IB, IF | Abcam (ab56416) |
| **YFP** (recognizes GFP) | RRID:AB_2313808 | IB | Clontech/Takara (632381) |

^a^ This is a replacement item for an antibody used in this study that is no longer commercially available. IB: Immunoblot; IF: immunofluorescence

**Table 3 |Small-interfering RNA sequences**

| **ON-TARGET plus SMARTpools siRNAs** | **Companies** |
| --- | --- |
|  |  |
| ***Setd2*** (human, SETD2 NM_014159) |  |
| UAAAGGAGGUAUAUCGAAU | Dharmacon (L-012448) |
| GAGAGGUACUCGAUCAUAA |  |
| GCUCAGAGUUAACGUUUGA |  |
| CCAAAGAUUCAGACAUAUA |  |
|  |  |
|  |  |
| **Non-targeting siRNA pool** |  |
| UGGUUUACAUGUCGACUAA | Dharmacon (D-001810) |
| UGGUUUACAUGUUGUGUGA |  |
| UGGUUUACAUGUUUUCUGA |  |
| UGGUUUACAUGUUUUCCUA |  |
|  |  |

**Table 4 |Primer sequences used in this study**

| **cDNA** (organism) | **Forward Primer** | **Reverse Primer** |
| --- | --- | --- |
| **Primers used for qPCR** | | |
| ***ACTB/β-actin***  (human) | gatcaagatcattgctcctc | ttgtcaagaaagggtgtaac |
| ***SETD2***  (human) | gaagagaagaatccctacaaag | caataatctccttgaccttgg |
| ***GAPDH***  (human) | acagttgccatgtagacc | tttttggttgagcacagg |
| ***ULK1***  (human) | tgcaccaccaattgcttagc | ggcatggactgtggtcatgag |
| ***MAP1LC3B***  (human) | gctcatcaagataatcagacg | gcataaaccatgtacaggaag |
| ***BECN1***  (human) | cagtatcagagagaatacagtg | tggaaggttgcattaaagac |
| ***PI3KC3***  (human) | gtactggagaaaatgaaactgg | ccaatatacaaaggttgctcc |
| ***PI3KR4***  (human) | ttacagccttctcctcatag | caacaacataggacctttctg |
| ***ATG7***  (human) | gattgtcctaaagcagttgg | cttttaggtccatacattcac |
| ***ATG9A***  (human) | cagtttgacactgaataccag | aaactcgagagaagaagagg |
| ***ATG14* (Both)**  (human) | aatttactcgagcagtgaag | ttagattcctgagggtatgc |
| ***ATG14* (Short)**  (human) | aaaatcccacgtgactggct | cgataaacctctcccggtcg |
| ***WIPI1***  (human) | tccagtggacacctttatatg | agctgtgggttttgattaag |
| ***LAMP2***  (human) | accaaagagcagactgtttc | cagctgtagaatactttccttg |
| ***SQSTM1/p62***  (human) | Sequence under closure RT2-qPCR Primer Assay Predesign (Qiagen) | |
