# Supplementary material for: SETD2 transcriptional control of ATG14L/S isoforms regulates autophagosome–lysosome fusion
Source: Cell Death Dis. 2022 Nov 12;13(11):953. doi: 10.1038/s41419-022-05381-9 (PMC9653477; doi:10.1038/s41419-022-05381-9)
Supplement: Supplementary file 2 — Supplementary data file 1 [file 41419_2022_5381_MOESM2_ESM.pdf]

Emsembl Gene: ATG14 ENSG00000126775

Chromosome 14: 55,366,366,391-55,411,830 reverse strand

ATG14-201 - ENST0000247178 with 10 exons encoding for ATG14L

ATG14-202 - ENST0000558189 with 7 exons encoding for ATG14S

Translated sequence / Flanking sequence / intron sequence / UTR

ATG transcription start site for *ATG14L* in exon 1 and *ATG14S* in alternative exon 4' are indicated.

Exon / Intron	Length	Sequence
5' upstream sequence		..........cagaatgcacccggttgaaaacaaaatcccacgtgactggctctcctctc
Exon 1<br>with ATG14L start site	229	AGGCCATCATGGCGTCTCCCAGTGGGAAGGGAGCCCGGGCGCTGGAGGCTCCTGGCTGCG<br>GGCCCCGGCCGCTCGCCCGGGACCTGGTGGACTCCGTGGACGATGCGGAGGGGCTGTACG<br>TGGCTGTGGAGCGCTGCCCGCTGTGCAACACTACCCGCCGGCGGCTGACCTGCGCCAAAT<br>GCGTTCAGAGCGGCGATTTCGTCTACTTCGACGGCCGCGACCGGGAGAG
Intron 1-2	14,167	gtacggcggccgccacggccctatt..........aagacattgaatttttttgttacag
Exon 2	63	GTTTATCGACAAGAAGGAAAGGTTAAGCCGACTTAAGAGCAAGCAAGAAGAATTTCAGAA<br>AGA
Intron 2-3	1,389	gtgagtgttttgttttgtcttttaa..........tattttctgtctcttttcctcacag
Exon 3	43	AGTGTTAAAAGCTATGGAAGGAAAATGGATAACAGATCAGTTG
Intron 3-4	4,947	gtaagttgtaatacatgttcttttt..........gatatttattacatgctttttttcag
5' upstream sequence		..........aggcatgcgccaccacgcccagctaatttttgtattttttttagtagagat
Exon 4'<br>with ATG14S start site		GGGGTTTCACCATGTTGGCCAGGATGGTCTTGATCTCCTAACCTCTTGATCTGCCCTCCT<br>TGGCCTCCCAAAGTGCTGCGATTACAGGCGTGAGCCACCGCACTGGCCTAATTTCTCTTT<br>TCTCTGCATTGAACTAGAAAAACATCGTTCTCTTTTTCGTAATGTTCCATAGTTACAAAA<br>GCTATAACATAAGGTCTTTTTCTTCATTATGTTTTCTGACATATCTGAAAATAAGCAGTG<br>ATCCCAGGTAGATAATCATATTAACCATAAGAGACCAACGTTTGTGATATTTATTACATG<br>CTTTTTTCAGAGATGGAAAATAATGTCCTGCAAGATGAGGATTGAACAGTTAAAACAAAC<br>AATATGTAAAGGAAATGAAGAAATGGAGAAAA
Exon 4	82	AGATGGAAAATAATGTCCTGCAAGATGAGGATTGAACAGTTAAAACAAACAATATGTAAA<br>GGAAATGAAGAAATGGAGAAAA
Intron 4-5	4,814	gtaagtaattcgtgtccttccttcc..........gttgggtgtgatttatttattttag
Exon 5	238	ATTCTGAAGGCCTTCTCAAAACCAAGGAAAAGAATCAGAAGCTTTACAGTCGAGCACAAC<br>GGCACCAAGAGAAAAAGGAGAAGATTCAGAGGCATAATCGCAAACTTGGTGACCTGGTAG<br>AAAAAAAGACCATTGACTTAAGAAGTCATTATGAGCGTCTGGCAAATCTTCGACGATCCC<br>ATATATTAGAGCTCACCTCTGTCATTTTTCCAATCGAGGAAGTAAAGACGGGTGTGAG
Intron 5-6	3,667	gtacattaagcaagtcttttgactc..........tccgtgtgtgtcttcctacttgtag
Exon 6	230	AGACCCCGCAGATGTGTCTTCAGAGAGTGACAGTGCCATGACCTCCAGCACTGTGAGCAA<br>GCTTGCTGAAGCCCGGAGGACAACTTACCTCTCAGGACGATGGGTCTGTGACGATCACAA<br>CGGAGACACCAGCATTAGCATTACAGGGCCTTGGATTAGCCTCCCTAACAATGGGGACTA<br>CTCTGCCTACTACAGCTGGGTGGAGGAGAAGAAAACAACCCAGGGGCCTG
Intron 6-7	1,271	gtgagaagcagcatatcctttgaaa..........catggtggttgttttcttactgcag
Exon 7	118	ACATGGAGCAGAGTAACCCTGCCTACACCATCAGTGCTGCGCTGTGCTATGCAACTCAGC<br>TGGTCAACATTCTGTCTCATATACTTGATGTAAATCTTCCCAAAAAGCTCTGCAACAG
Intron 7-8	2,498	gcaagtaattgaaggtggtaatgtc..........tataaattgtatgtttgttttacag
Exon 8	91	TGAATTTTGTGGCGAAAATCTAAGCAAGCAGAAATTTACTCGAGCAGTGAAGAAACTGAA<br>TGCAAATATTCTTTACCTTTGTTTTTCTCAG
Intron 8-9	79	gtatgaaaaataggttgtgaactaa..........taaccaattctttaatttttttag
Exon 9	86	CATGTAAATTTAGATCAATTACAACCACTGCATACCCTCAGGAATCTAATGTACCTGGTC<br>AGTCCAAGCTCTGAACACCTAGGCAG

Exon / Intron	Length	Sequence
Intron 9-10	7,893	gtaagaagatatttgttgcttttct..........agagaccctcattgtctgcacacag
Exon 10	3,535	GTCAGGGCCCTTTGAAGTACGAGCAGACCTTGAGGAGTCCATGGAATTTGTGGATCCCGG<br>AGTTGCTGGAGAATCAGATGAGAGCGGAGATGAGCGCGTCAGCGATGAAGAAACCGACCT<br>GGGCACAGACTGGGAGAACTTGCCTAGTCCCCGGTTTTGTGATATCCCTTCCCAGTCTGT<br>GGAAGTCTCCCAGAGTCAGAGCACCCAGGCGTCCCCACCCATCGCGAGCAGCAGTGCAGG<br>TGGGATGATCTCCTCTGCAGCAGCCTCGGTGACCTCCTGGTTTAAAGCTTACACTGGACA<br>CCGTTAACGAGCATGGACCAAAACATACCAAATCTGCATCAAGAAAGTTCTTCTCCCACT<br>ACACTCTAGTAAACATTTTCTGTTTAAGTTAAGATAGTGTCTGGAACAAAGAGGTTAAAG<br>TGTTGTTTTGTTTTGTCTTTTTAAGCAGGGAGACAAACATTTCTATTTGCCAAGTGGCCT<br>GTGATGGTGACCAACATGCTTATGATAATTAAGAGAACAGGGGTCGAAGGTCTTTCTACC<br>CAGACCAGTGCTGGTGGAAGGAGGACCTGTGCGTGTGGCCAGTTCTGCCAAGGAAGCAGT<br>TGATTTGGGTTCCCTCTGGGCCCGGGCCACCGGGCCCACAGATATGGGTCAGTGTGCTGG<br>TCCTTGCGGTGCTGAGACTGTTCCTGACACTTTAAGTTTTAGAGGTTGGTTGAATCACAA<br>GAGGTGATTCTTGATTATTAGGACATGAAAGATAAAAGCTCTTTAATAAGAGTTTTTCTG<br>CCATTGTTTTTTGTATGAGAACCAGCAGGCAATTTAAAATTTCTAATTTGGTCCTTTGAT<br>TTTGTTTGGGAGGGGTGAGTTACACGTATTTTATTCATGCTGCTCTGTCGTAGTTTGTCA<br>GACATTCCTGTTTTTCTTTCCCCCACACACCAAAGAAAATGAAAGTCTTTTTCTTTAGGA<br>CCCACATCCATAAATGGAAGAAATCCTGGCTGCAATAATGTCTAGAGAGTTTTTAACTAT<br>TTTCTTGTATTCTGAGGGGAATTAAGCTTATTCTTACCTAGTTGAATTCCTGCCATCCAC<br>ACTATGAGCATTTTGAAATTGAACTTATATTTTCTGGGTGAAAATAAGTCATGAAGGTCA<br>TTCCCTTATGTAAGCTCAATGCCTGCCTGGGCACAGGGGAAAAGCCACTTAGTTAAGTGG<br>CCTCTGGTCATTCTTGTGGTGTCCACTTTCTTTCTATGGGATTGAGTAGGTGGCAGGTGT<br>TTTCAGGGGAAACCATCCTACTTGTTTCCCCGAACTCTTTGTTGCTCTGAGGACACAGCT<br>TTGCTCAGAAATGCAGCGCAGATCCTTACGGCTGATGCTACTCTGCTCTGTTCTGGGGAA<br>AGCACAATATAAAGAAAGAATTTCCCAGCCAGGCGCAGTGGCTCACGCCTGTAATCCCAG<br>CACTTTAGGAGGCCGAGGCAGGCGGATCACTTGAGGTCAGGAGTTTGAGAGCAGCCTGGC<br>TAACATGGTGAAACCCTGTTTCTACTAAAAATACAAAAAATTACCGGGTGTGGTGGCGCA<br>CGCCTGTAATCCCAGCTACTCGGGAGGCTGCGGCAGGAGAATCGCTTGAACCGGGAGGCA<br>GAGGTTGCAGTGAGCCGAGATTGTGCCATTGCACTCCAGCCTGGGCAACAAGAGCGAAAC<br>TCCGTCTCAAAAAAAAAAAGAATTTCCCTCAGCAGGAGATCATTTTCAGCTCACGTGTCT<br>TGTCATTCTTTTAGTGACAATCTTACAAGAAAACTATAATGAGAGAGGCATTATGTACAA<br>ATATGTAAGTAGTTTATTTTTAATAACTGCAAAAAAATCCTATGTAACAACTACCAAAAG<br>AAATCCTATGAAAGAGTCCTAACAGGCATTATTACCATATCTTATGTGATTGGCATGATA<br>GCACCTCTGATAAATCATTCAGAGGTTTGCCATGCCCCAGCTTCTTTTCTCATCATAATA<br>ATTGTAGTTGATACTTTGCCTCCAAGTCCGAGGTGCTATATAGCTTTTGCTAATGGTATA<br>TTTGGTGTTTTGTATAGTTTTGGGTAGAGTTGCAGAACGGAGTTTATTTCTATCCGGTAG<br>TCACAAATTCCTTGGCTCTATGAATTTTCCATGAAAGGAGGAAGTAGGCTTTTCTCGTTG<br>TGGGTGGTCTTTTTTTTTTTTGGAGACGGAGTCTCACTCAGCTGCCCAGGCTGGAGTGTA<br>GTGGCACCATCTCCGCTCACTGCAACCACCATCTCCTGGGTTCAAGCAATTCTTCCATCT<br>CAACCTCCCGAGTAGCTGGGATTATAGGCACCTGCCATCATGCCCAGCTAATTTTTGTAT<br>TTTAGTAAAGACGGGGGTTTTCACCATGTTGGCCAGGCTGGTCTTGAACTCCTGACTTCA<br>GGTGATCCGCTTGCCTTGGCCTCCTAAAGTGCTAGGATTACAGGCCTGAGCCACCGCGCC<br>CGGCCCCTTATGGGTTCTTCTACACTGCTGGGATCTCTGTTTTAAGTGCTCAGCTTCATG<br>ATTGATTGCTGGGCTTCCATTTTCCCATCCAGTTCTGGAGTTCGTAGAGAGTGAAGATGG<br>TAGACTTGAACAGATAAATAAACTTAACGATCTTGTAAGAGTTGTCTAGCTACTTAAAAC<br>CCTCAGAAGTAAGAGCTTAGTCTCACGAGTTGTAAGAGTGGGATTTGGAGCTTGGTGGTG<br>GAGACTGACTTCAGCTGAGAGATGCACAACAGTCATGGTTTTCTTAAGCCTCTTATGAAA<br>CCATGAATGAGAGATGAAGCTAAAGAATAGAATCCAGAGATCACAAACTCATCTAGAGTA<br>CTTCCACAAAATTTACAAAGATGTGGGAACTTTATGGATAGGATATATTTTGTTTGTTGT<br>TGTTAATATCAACTAGAGGCACTTTACATAGGGTTAAGTGATCGAACCCTTTTGTGGTTT<br>TGAACACCAACATACTGGCTTACACTGCTGAAATATTTTGGGTTTCATTATTTTGCACTG<br>GATCCACCCTGTAAATACTCTTAAGTATACATTTCAACCACTGTTTTTTCTACTCTTTTT<br>GCTGCTCATTAAAATCTTTCATGTAGGTGCCAGAACCATATGTAAACAGCTTTTTAAAAA<br>ATTGAAGCTGGTATTTTGTTTAAACAAAAAGCCATAGAACTTGGTCATGTTTTCCATTTT<br>AAAATGATTTACTGAAACAAAGTAATACTAATAAAAACCCACAGGCACCAAACAGGCTGC<br>TTAAAATGGTCTGTTAAAGACATTTTTTGGTTATGGAATATAAGAAAAGTTTTGCACATC<br>TGTAAGGGGGAAAAACAGTATATCACCATTGGGTAGAGTGGACGGGACTCATGTAAGGAC<br>TCAATTTGGGGAAGAGCATTCAGTGGCATGCTGTTAGAGGACTAGTGTCCGAGAATCTCC<br>TCACAGTATCATGTTGCAGGAATTCCCCATTGCTCTGCAACTTCCAAACCAGTTTGAGTC<br>ATACAAATGTTTTCTAAACTTTTATTGTATTACTGCAATAAATCTTTTAACAGTA
3' downstream sequence		ctttgctcatgtgtatgtctttttcaaactgttcatttgcactgctgcat..........
