# Supplementary material for: SETD2 transcriptional control of ATG14L/S isoforms regulates autophagosome–lysosome fusion
Source: Cell Death Dis. 2022 Nov 12;13(11):953. doi: 10.1038/s41419-022-05381-9 (PMC9653477; doi:10.1038/s41419-022-05381-9)

Figure 1a

Atg8

Pgk1

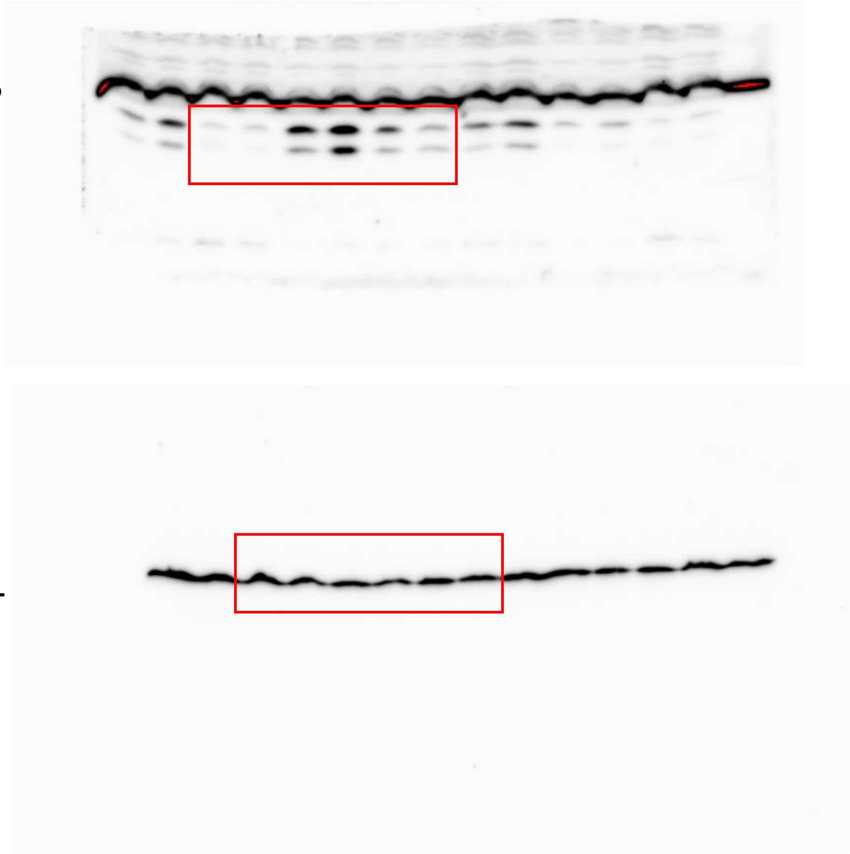

Figure 1c

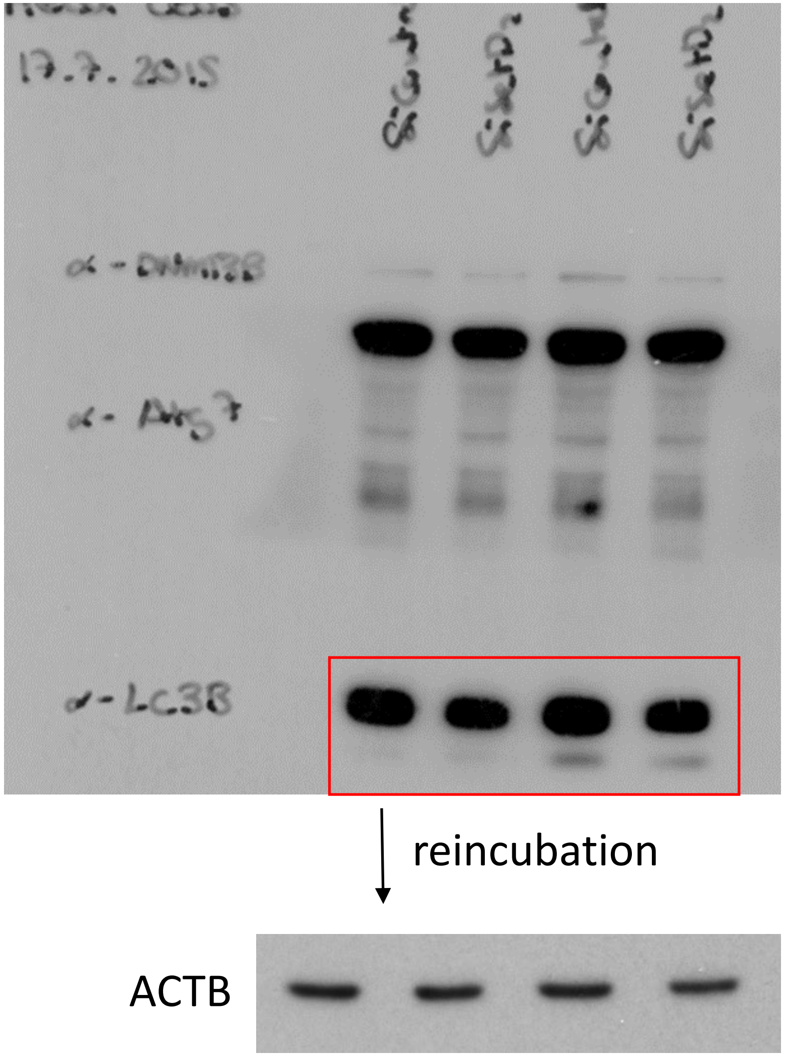

Figure 1f

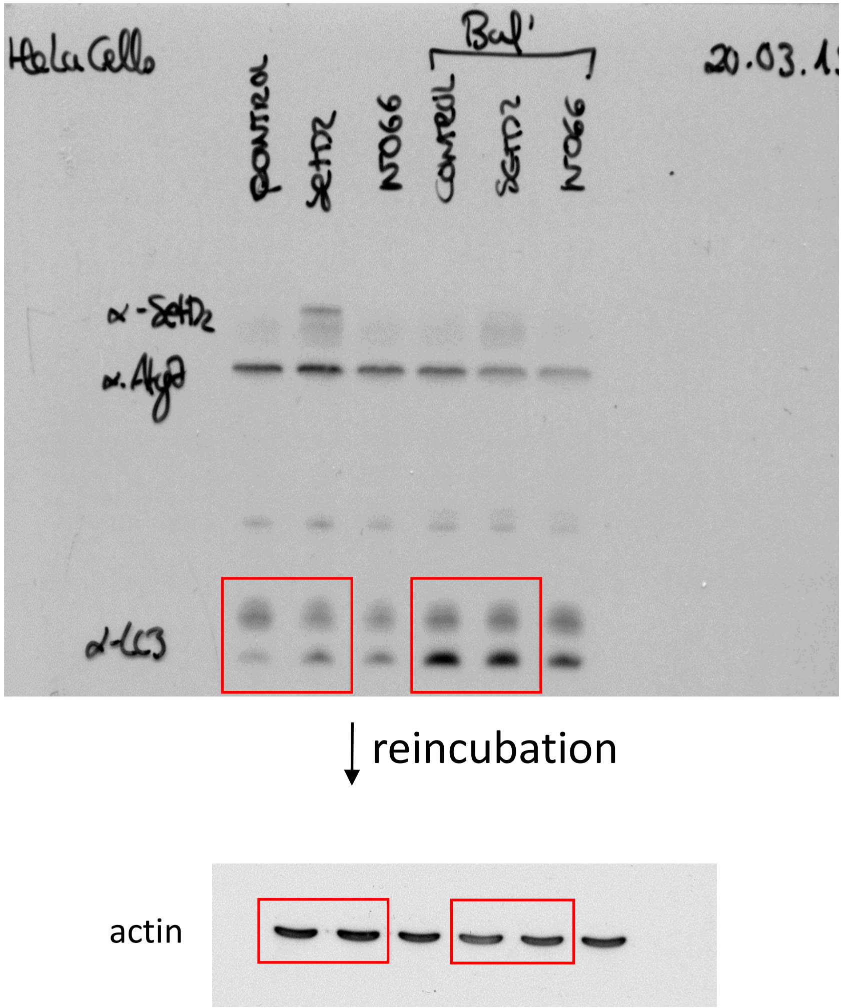

Figure 4a

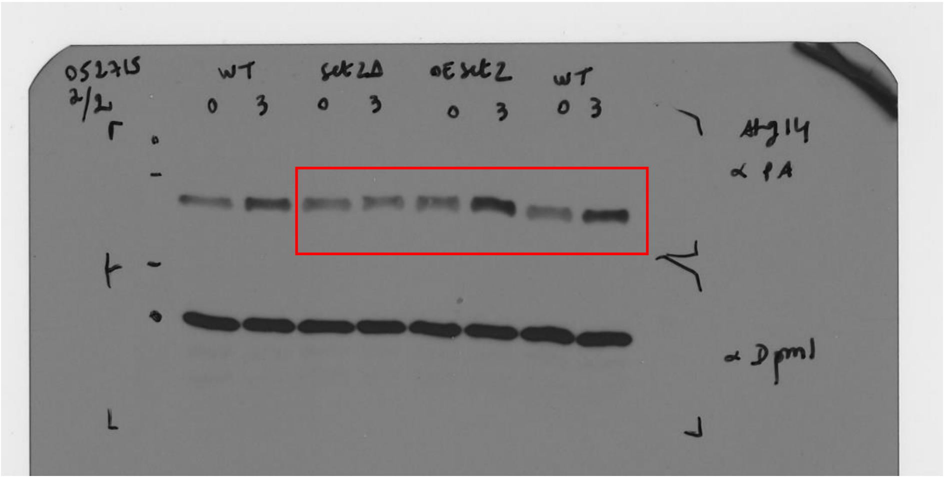

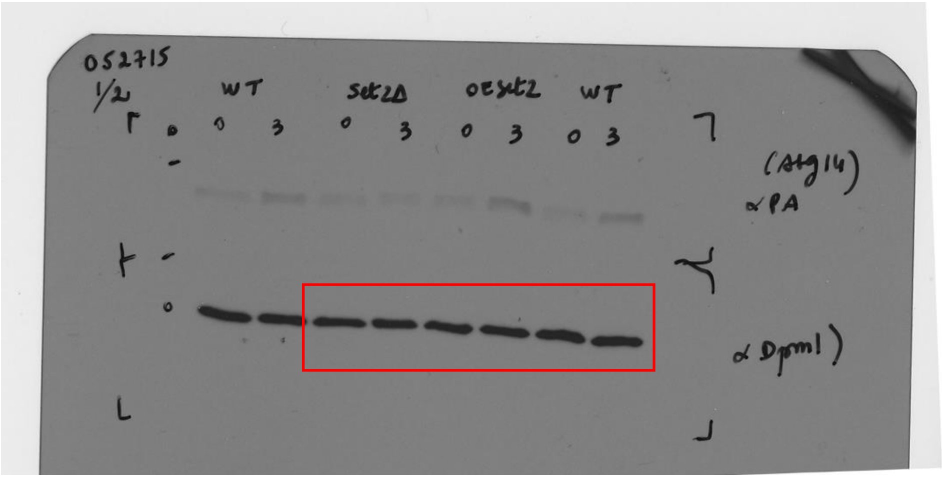

Figure 4c

ATG14

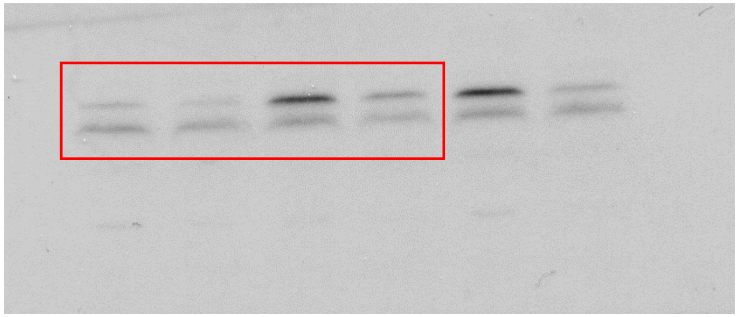

reincubation

ACTB

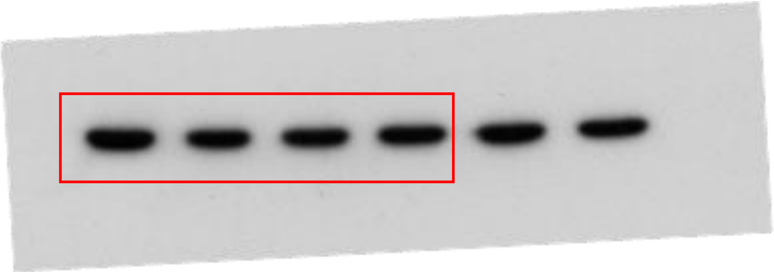

Figure 6c

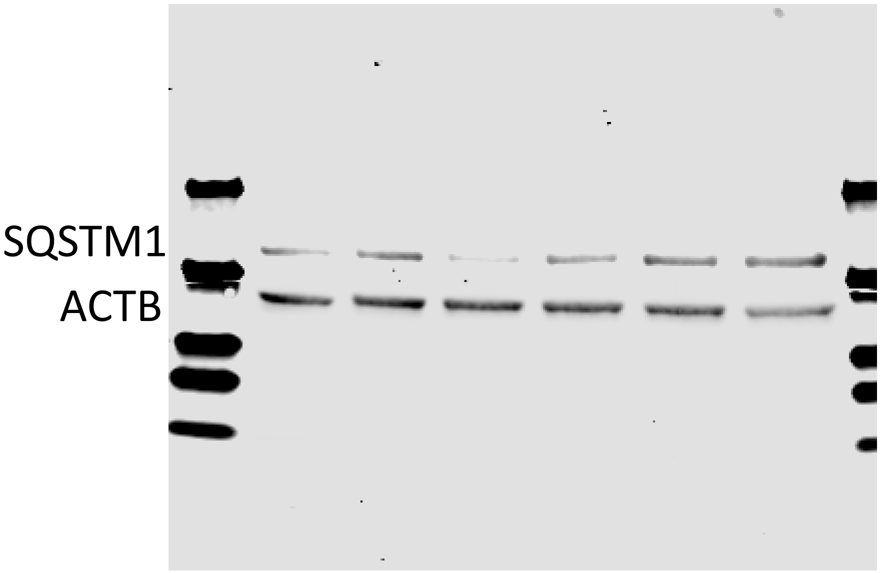

Figure 6f

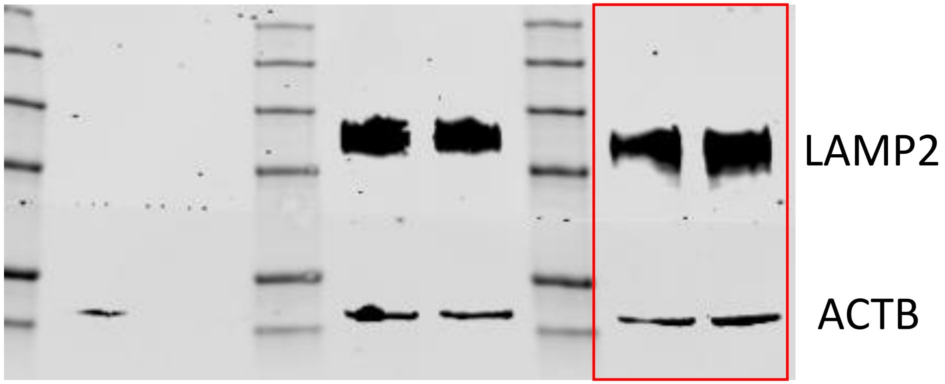

Figure 7c

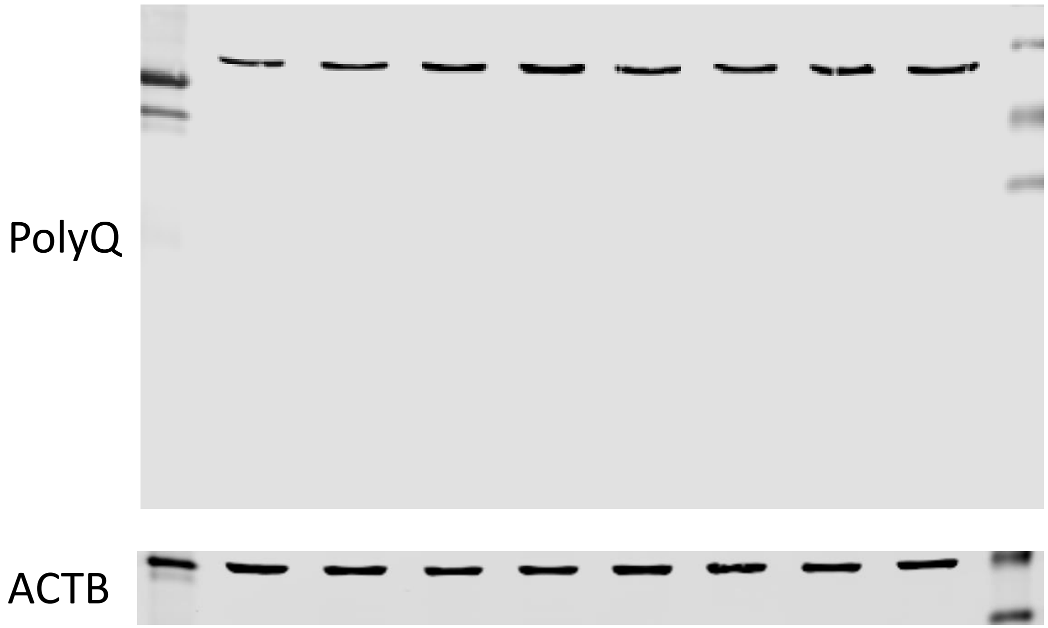

Supplementary figure S1b

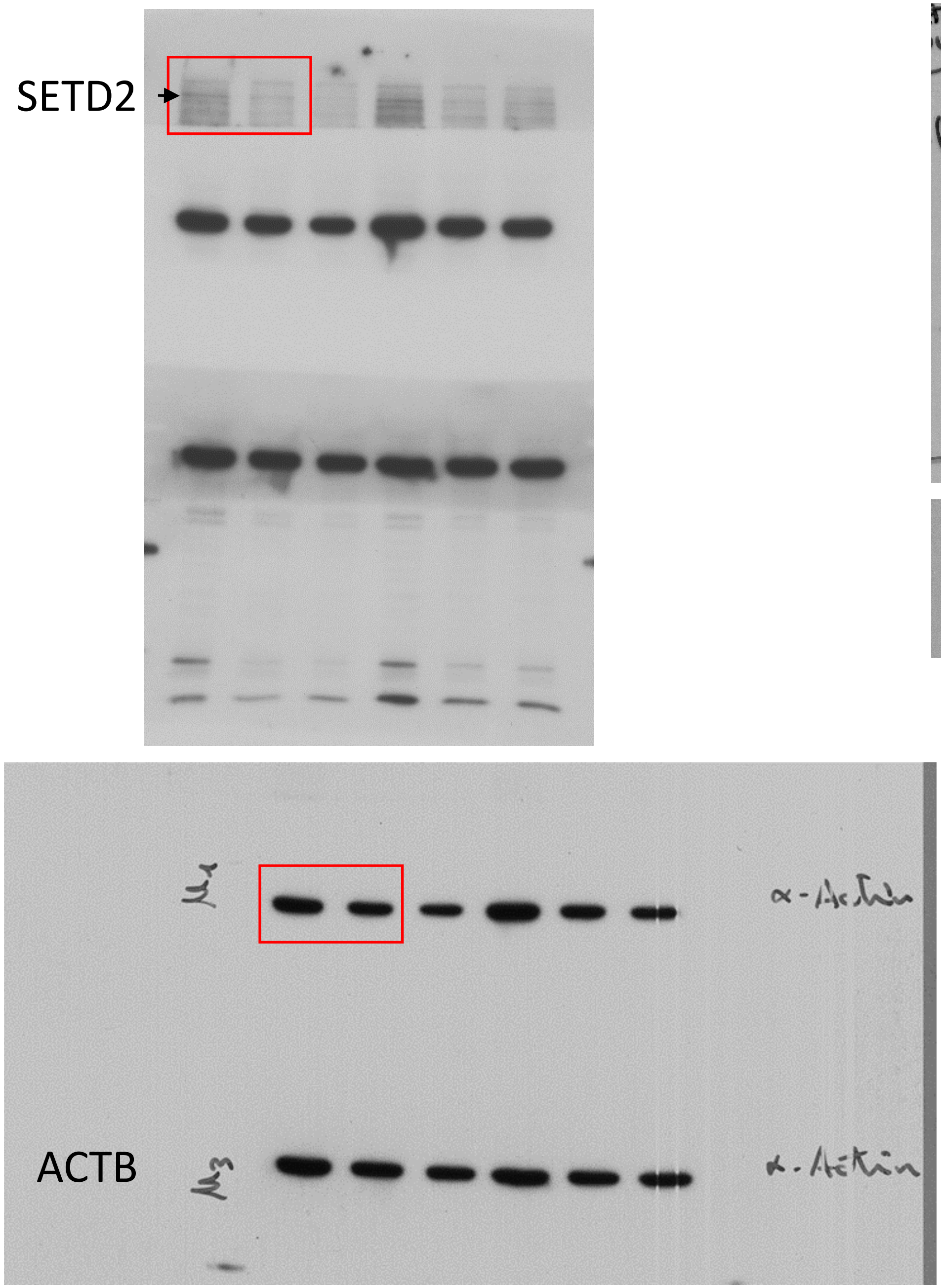

Supplementary figure S1d

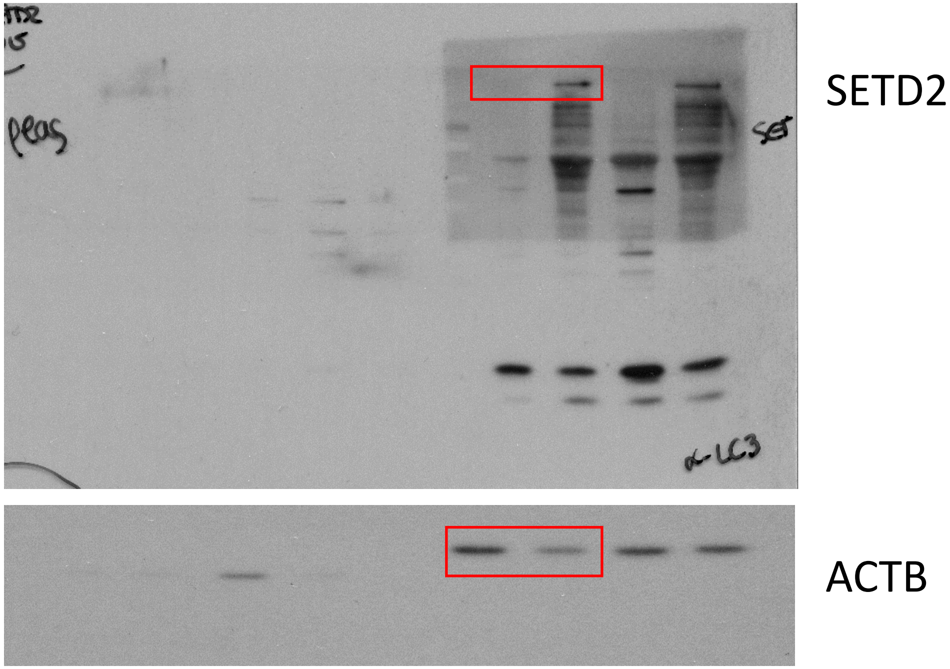

Supplementary figure S3a

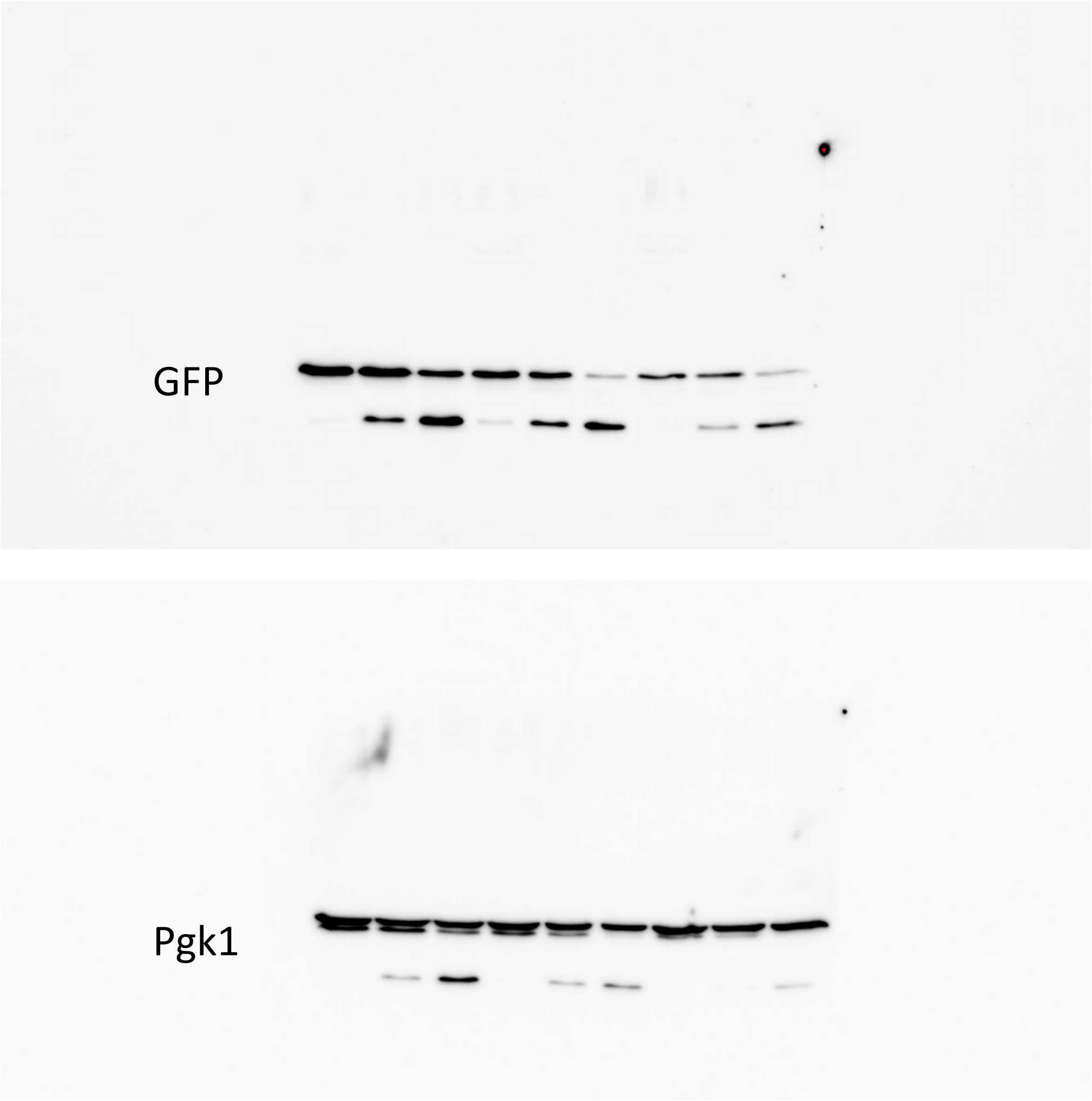

Supplement: Supplementary file 3 — Uncuted Immunoblots [file 41419_2022_5381_MOESM3_ESM.pdf]
