# Supplementary material for: SETD2 transcriptional control of ATG14L/S isoforms regulates autophagosome–lysosome fusion
Source: Cell Death Dis. 2022 Nov 12;13(11):953. doi: 10.1038/s41419-022-05381-9 (PMC9653477; doi:10.1038/s41419-022-05381-9)
Supplement: Supplementary file 5 — Authors' agreement [file 41419_2022_5381_MOESM5_ESM.pdf]

Re: CDDIS-22-3172RR Initial Quality Check

Patricia Gonzalez-Rodriguez <patricia.gonzalez-rodriguez@medisin.uio.no>
Tue 10/25/2022 10:26
To: Bertrand Joseph <bertrand.joseph@ki.se>

Hi Bertrand,

I agree to include Pinelopi as author for her contribution to the paper.

Best regards,

Patricia

From: Bertrand Joseph <bertrand.joseph@ki.se>
Sent: 25 October 2022 10:23:19
To: Patricia Gonzalez-Rodriguez; Elizabeth Delorme-Axford; amelie.bernard@u-bordeaux.fr; Lily Keane; Vassilis Stratoulias; Kathleen Grabert; Pinelopi Engskog Vlachos; Jens Füllgrabe; Dan Klionsky
Subject: Fw: CDDIS-22-3172RR Initial Quality Check

Dear Patricia, Elizabeth, Amelie, Lily, Vassilis, Kathleen, Poppi, Jens, and Dan,

Pinelopi Engskog-Vlachos in our laboratory who contributed to the generation of the new data set included during the revision of our manuscript entitled "SETD2 transcriptional control of ATG14L/S isoforms regulates autophagosome-lysosome fusion" to *Cell Death & Disease* has been added as co-author for her contribution.

The list of authors is as follows:
Patricia González-Rodríguez, Elizabeth Delorme-Axford, Amelie Bernard, Lily Keane, Vassilis Stratoulias, Kathleen Grabert, Pinelopi Engskog-Vlachos, Jens Füllgrabe, Daniel J. Klionsky, Bertrand Joseph.

Could you please all (including Poppi) confirm by return of email that you agree to this change in the list of authors? See request from the journal below.

I take the chance to thank you for our long-term successful and stimulating collaboration.

best regards

Bertrand

Bertrand Joseph | PhD
Professor of Molecular Cancer Biology

Institute of Environmental Medicine | Karolinska Institutet
Toxicology Unit, Nobelsväg 13
SE-171 77 Stockholm, Sweden
bertrand.joseph@ki.se | Joseph's Group | Karolinska Institutet

Karolinska Institutet is one of the world's leading medical universities. Its mission is to contribute to the improvement of human health through research and education. Karolinska Institutet accounts for over 40 per cent of the medical academic research conducted in Sweden and offers the country's broadest range of education in medicine and health sciences. Since 1901 the Nobel Assembly at Karolinska Institutet has selected the Nobel laureates in Physiology or Medicine.

From: cddisease@springernature.com <cddisease@springernature.com>
Sent: Monday, October 24, 2022 17:52
To: Bertrand Joseph <bertrand.joseph@ki.se>
Subject: CDDIS-22-3172RR Initial Quality Check

Dear Professor Joseph,

In checking in your manuscript submitted to Cell Death & Disease it has come to our attention that the following must be addressed before we can proceed.

1. It has come to our attention that your most recent author list differs from the one in your original submission. We find that the following authors have been added since your initial submission: Pinelopi Engskog-Vlachos

Please request agreement from all authors including additions and deletions, these can be collected in the following way:

Email your co-authors with the change, and ask them to reply to your email confirming that they agree to these changes. Once you have collected these replies, please combine all of the co-authors' email responses in one document and upload this file to your submission.

Your paper is now available for you to edit, you may access via the following link:

Please make the correction(s) as specified above and resubmit your paper following the same steps as before.

If you have any questions please feel free to contact us.

Sincerely,

Editorial Office
Cell Death & Disease
cddisease@springernature.com

This email has been sent through the NPG Manuscript Tracking System NY-610A-NPG&MTS

Confidentiality Statement:

This e-mail is confidential and subject to copyright. Any unauthorised use or disclosure of its contents is prohibited. If you have received this email in error please notify our Manuscript Tracking System Helpdesk team at http://platformsupport.nature.com .
Details of the confidentiality and pre-publicity policy may be found here http://www.nature.com/authors/policies/confidentiality.html
Privacy Policy | Update Profile

När du skickar e-post till Karolinska Institutet (KI) innebär detta att KI kommer att behandla dina personuppgifter. Här finns information om hur KI behandlar personuppgifter.

Sending email to Karolinska Institutet (KI) will result in KI processing your personal data. You can read more about KI's processing of personal data here.

Re: CDDIS-22-3172RR Initial Quality Check

Elizabeth Delorme-Axford <delormeaxford@oakland.edu>
Tue 10/25/2022 18:24
To: Bertrand Joseph <bertrand.joseph@ki.se>
Cc: Jens Füllgrabe <jensfullgrabe@gmail.com>;Kathleen Grabert <kathleen.grabert@ki.se>;Lily Keane <lily.keane@ki.se>;Pinelopi Engskog Vlachos <pinelopi.vlachos@ki.se>;Patricia Gonzalez-Rodriguez (UIO) <patricia.gonzalez-rodriguez@medisin.uio.no>;amelie.bernard@u-bordeaux.fr <amelie.bernard@u-bordeaux.fr>;Vassilis Stratoulias <vassilis.stratoulias@ki.se>;Dan Klionsky <klionsky@umich.edu>

Yes, I agree.

Beth

On Tue, Oct 25, 2022 at 7:29 AM Bertrand Joseph <bertrand.joseph@ki.se> wrote:

> Thanks,
> Bertrand
>
> ---
>
> **From:** Jens Füllgrabe <jensfullgrabe@gmail.com>
> **Sent:** Tuesday, October 25, 2022 13:27
> **To:** Bertrand Joseph <bertrand.joseph@ki.se>
> **Cc:** Kathleen Grabert <kathleen.grabert@ki.se>; Lily Keane <lily.keane@ki.se>; Pinelopi Engskog Vlachos <pinelopi.vlachos@ki.se>; Patricia Gonzalez-Rodriguez (UIO) <patricia.gonzalez-rodriguez@medisin.uio.no>; Elizabeth Delorme-Axford <delormeaxford@oakland.edu>; amelie.bernard@u-bordeaux.fr <amelie.bernard@u-bordeaux.fr>; Vassilis Stratoulias <vassilis.stratoulias@ki.se>; Dan Klionsky <klionsky@umich.edu>
> **Subject:** Re: CDDIS-22-3172RR Initial Quality Check
>
> Hi,
>
> Yes, of course.
>
> Kind regards,
> Jens
>
> On Tue, 25 Oct 2022, 11:29 Bertrand Joseph, <bertrand.joseph@ki.se> wrote:
>
>> Great, thanks!
>>
>> ---
>>
>> **From:** Kathleen Grabert <kathleen.grabert@ki.se>
>> **Sent:** Tuesday, October 25, 2022 10:59
>> **To:** Lily Keane <lily.keane@ki.se>; Bertrand Joseph <bertrand.joseph@ki.se>; Pinelopi Engskog Vlachos <pinelopi.vlachos@ki.se>; Patricia Gonzalez-Rodriguez (UIO) <patricia.gonzalez-rodriguez@medisin.uio.no>; Elizabeth Delorme-Axford <delormeaxford@oakland.edu>; amelie.bernard@u-bordeaux.fr <amelie.bernard@u-bordeaux.fr>; Vassilis Stratoulias <vassilis.stratoulias@ki.se>; Jens Füllgrabe <jensfullgrabe@gmail.com>; Dan Klionsky <klionsky@umich.edu>
>> **Subject:** RE: CDDIS-22-3172RR Initial Quality Check
>>
>> It's a YES from me!

From: Lily Keane <lily.keane@ki.se>
Sent: den 25 oktober 2022 10:36
To: Bertrand Joseph <bertrand.joseph@ki.se>; Pinelopi Engskog Vlachos <pinelopi.vlachos@ki.se>; Patricia Gonzalez-Rodriguez (UIO) <patricia.gonzalez-rodriguez@medisin.uio.no>; Elizabeth Delorme-Axford <delormeaxford@oakland.edu>; amelie.bernard@u-bordeaux.fr; Vassilis Stratoulias <vassilis.stratoulias@ki.se>; Kathleen Grabert <kathleen.grabert@ki.se>; Jens Füllgrabe <jensfullgrabe@gmail.com>; Dan Klionsky <klionsky@umich.edu>
Subject: Re: CDDIS-22-3172RR Initial Quality Check

Agree

Get Outlook for Android

From: Bertrand Joseph <bertrand.joseph@ki.se>
Sent: Tuesday, October 25, 2022 10:34:05 AM
To: Pinelopi Engskog Vlachos <pinelopi.vlachos@ki.se>; Patricia Gonzalez-Rodriguez (UIO) <patricia.gonzalez-rodriguez@medisin.uio.no>; Elizabeth Delorme-Axford <delormeaxford@oakland.edu>; amelie.bernard@u-bordeaux.fr <amelie.bernard@u-bordeaux.fr>; Lily Keane <lily.keane@ki.se>; Vassilis Stratoulias <vassilis.stratoulias@ki.se>; Kathleen Grabert <kathleen.grabert@ki.se>; Jens Füllgrabe <jensfullgrabe@gmail.com>; Dan Klionsky <klionsky@umich.edu>
Subject: Re: CDDIS-22-3172RR Initial Quality Check

Thank you!

From: Pinelopi Engskog Vlachos <pinelopi.vlachos@ki.se>
Sent: Tuesday, October 25, 2022 10:31
To: Bertrand Joseph <bertrand.joseph@ki.se>; Patricia Gonzalez-Rodriguez (UIO) <patricia.gonzalez-rodriguez@medisin.uio.no>; Elizabeth Delorme-Axford <delormeaxford@oakland.edu>; amelie.bernard@u-bordeaux.fr <amelie.bernard@u-bordeaux.fr>; Lily Keane <lily.keane@ki.se>; Vassilis Stratoulias <vassilis.stratoulias@ki.se>; Kathleen Grabert <kathleen.grabert@ki.se>; Jens Füllgrabe <jensfullgrabe@gmail.com>; Dan Klionsky <klionsky@umich.edu>
Subject: Sv: CDDIS-22-3172RR Initial Quality Check

Hi Bertrand

I agree to this change in the list of authors.

Best

Pinelopi Engskog Vlachos

Från: Bertrand Joseph <bertrand.joseph@ki.se>
Skickat: den 25 oktober 2022 10:23
Till: Patricia Gonzalez-Rodriguez (UIO) <patricia.gonzalez-rodriguez@medisin.uio.no>; Elizabeth Delorme-Axford <delormeaxford@oakland.edu>; amelie.bernard@u-bordeaux.fr; Lily Keane <lily.keane@ki.se>; Vassilis Stratoulias <vassilis.stratoulias@ki.se>; Kathleen Grabert <kathleen.grabert@ki.se>; Pinelopi Engskog Vlachos <pinelopi.vlachos@ki.se>; Jens Füllgrabe <jensfullgrabe@gmail.com>; Dan Klionsky <klionsky@umich.edu>
Ämne: Fw: CDDIS-22-3172RR Initial Quality Check

Dear Patricia, Elizabeth, Amelie, Lily, Vassilis, Kathleen, Poppi, Jens, and Dan,

Pinelopi Engskog-Vlachos in our laboratory who contributed to the generation of the new data set included during the revision of our manuscript entitled "SETD2 transcriptional control of ATG14L/S isoforms regulates autophagosome-lysosome fusion" to *Cell Death & Disease* has been added as co-author for her contribution.

The list of authors is as follows:

Patricia González-Rodríguez, Elizabeth Delorme-Axford, Amelie Bernard, Lily Keane, Vassilis Stratoulias, Kathleen Grabert, Pinelopi Engskog-Vlachos, Jens Füllgrabe, Daniel J. Klionsky, Bertrand Joseph.

Could you please all (including Poppi) confirm by return of email that you agree to this change in the list of authors? See request from the journal below.

I take the chance to thank you for our long-term successful and stimulating collaboration.

best regards

Bertrand

Bertrand Joseph | PhD
Professor of Molecular Cancer Biology

Institute of Environmental Medicine | Karolinska Institutet
Toxicology Unit, Nobelsväg 13

SE-171 77 Stockholm, Sweden
bertrand.joseph@ki.se | Joseph's Group | Karolinska Institutet

---

Karolinska Institutet is one of the world's leading medical universities. Its mission is to contribute to the improvement of human health through research and education. Karolinska Institutet accounts for over 40 per cent of the medical academic research conducted in Sweden and offers the country's broadest range of education in medicine and health sciences. Since 1901 the Nobel Assembly at Karolinska Institutet has selected the Nobel laureates in Physiology or Medicine.

From: cddisease@springernature.com <cddisease@springernature.com>
Sent: Monday, October 24, 2022 17:52
To: Bertrand Joseph <bertrand.joseph@ki.se>
Subject: CDDIS-22-3172RR Initial Quality Check

Dear Professor Joseph,

In checking in your manuscript submitted to Cell Death & Disease it has come to our attention that the following must be addressed before we can proceed.

1. It has come to our attention that your most recent author list differs from the one in your original submission. We find that the following authors have been added since your initial submission: Pinelopi Engskog-Vlachos

Please request agreement from all authors including additions and deletions, these can be collected in the following way:

Email your co-authors with the change, and ask them to reply to your email confirming that they agree to these changes. Once you have collected these replies, please combine all of the co-authors’ email responses in one document and upload this file to your submission.

Your paper is now available for you to edit, you may access via the following link:

Please make the correction(s) as specified above and resubmit your paper following the same steps as before.

If you have any questions please feel free to contact us.

Sincerely,

Editorial Office
Cell Death & Disease
cddisease@springernature.com

This email has been sent through the NPG Manuscript Tracking System NY-610A-NPG&MTS

Confidentiality Statement:

This e-mail is confidential and subject to copyright. Any unauthorised use or disclosure of its contents is prohibited. If you have received this email in error please notify our Manuscript Tracking System Helpdesk team at http://platformsupport.nature.com .

Details of the confidentiality and pre-publicity policy may be found here http://www.nature.com/authors/policies/confidentiality.html

Privacy Policy | Update Profile

När du skickar e-post till Karolinska Institutet (KI) innebär detta att KI kommer att behandla dina personuppgifter. Här finns information om hur KI behandlar personuppgifter.

Sending email to Karolinska Institutet (KI) will result in KI processing your personal data. You can read more about KI's processing of personal data here.

Re: CDDIS-22-3172RR Initial Quality Check

Amélie Bernard <amelie.bernard@u-bordeaux.fr>
Tue 10/25/2022 11:54
To: Kathleen Grabert <kathleen.grabert@ki.se>
Cc: Lily Keane <lily.keane@ki.se>;Bertrand Joseph <bertrand.joseph@ki.se>;Pinelopi Engskog Vlachos <pinelopi.vlachos@ki.se>;Patricia Gonzalez-Rodriguez, UIO <patricia.gonzalez-rodriguez@medisin.uio.no>;Elizabeth Delorme-Axford <delormeaxford@oakland.edu>;Vassilis Stratoulias <vassilis.stratoulias@ki.se>;Jens Füllgrabe <jensfullgrabe@gmail.com>;Dan Klionsky <klionsky@umich.edu>

Yes, of course!

Amélie

De: "Kathleen Grabert" <kathleen.grabert@ki.se>
À: "Lily Keane" <lily.keane@ki.se>, "Bertrand Joseph" <bertrand.joseph@ki.se>, "Pinelopi Engskog Vlachos" <pinelopi.vlachos@ki.se>, "Patricia Gonzalez-Rodriguez, UIO" <patricia.gonzalez-rodriguez@medisin.uio.no>, "Elizabeth Delorme-Axford" <delormeaxford@oakland.edu>, "Amélie Bernard" <amelie.bernard@u-bordeaux.fr>, "Vassilis Stratoulias" <vassilis.stratoulias@ki.se>, "Jens Füllgrabe" <jensfullgrabe@gmail.com>, "Dan Klionsky" <klionsky@umich.edu>
Envoyé: Mardi 25 Octobre 2022 10:59:40
Objet: RE: CDDIS-22-3172RR Initial Quality Check

It's a YES from me!

From: Lily Keane <lily.keane@ki.se>
Sent: den 25 oktober 2022 10:36
To: Bertrand Joseph <bertrand.joseph@ki.se>; Pinelopi Engskog Vlachos <pinelopi.vlachos@ki.se>; Patricia Gonzalez-Rodriguez (UIO) <patricia.gonzalez-rodriguez@medisin.uio.no>; Elizabeth Delorme-Axford <delormeaxford@oakland.edu>; amelie.bernard@u-bordeaux.fr; Vassilis Stratoulias <vassilis.stratoulias@ki.se>; Kathleen Grabert <kathleen.grabert@ki.se>; Jens Füllgrabe <jensfullgrabe@gmail.com>; Dan Klionsky <klionsky@umich.edu>
Subject: Re: CDDIS-22-3172RR Initial Quality Check

Agree

Get Outlook for Android

From: Bertrand Joseph <bertrand.joseph@ki.se>
Sent: Tuesday, October 25, 2022 10:34:05 AM
To: Pinelopi Engskog Vlachos <pinelopi.vlachos@ki.se>; Patricia Gonzalez-Rodriguez (UIO) <patricia.gonzalez-rodriguez@medisin.uio.no>; Elizabeth Delorme-Axford <delormeaxford@oakland.edu>; amelie.bernard@u-bordeaux.fr <amelie.bernard@u-bordeaux.fr>; Lily Keane <lily.keane@ki.se>; Vassilis Stratoulias <vassilis.stratoulias@ki.se>; Kathleen Grabert <kathleen.grabert@ki.se>; Jens Füllgrabe <jensfullgrabe@gmail.com>; Dan Klionsky <klionsky@umich.edu>
Subject: Re: CDDIS-22-3172RR Initial Quality Check

Thank you!

From: Pinelopi Engskog Vlachos <pinelopi.vlachos@ki.se>
Sent: Tuesday, October 25, 2022 10:31
To: Bertrand Joseph <bertrand.joseph@ki.se>; Patricia Gonzalez-Rodriguez (UIO) <patricia.gonzalez-

rodriguez@medisin.uio.no>; Elizabeth Delorme-Axford <delormeaxford@oakland.edu>; amelie.bernard@u-bordeaux.fr <amelie.bernard@u-bordeaux.fr>; Lily Keane <lily.keane@ki.se>; Vassilis Stratoulias <vassilis.stratoulias@ki.se>; Kathleen Grabert <kathleen.grabert@ki.se>; Jens Füllgrabe <jensfullgrabe@gmail.com>; Dan Klionsky <klionsky@umich.edu>
Subject: Sv: CDDIS-22-3172RR Initial Quality Check

Hi Bertrand
I agree to this change in the list of authors.

Best
Pinelopi Engskog Vlachos

Från: Bertrand Joseph <bertrand.joseph@ki.se>
Skickat: den 25 oktober 2022 10:23
Till: Patricia Gonzalez-Rodriguez (UIO) <patricia.gonzalez-rodriguez@medisin.uio.no>; Elizabeth Delorme-Axford <delormeaxford@oakland.edu>; amelie.bernard@u-bordeaux.fr; Lily Keane <lily.keane@ki.se>; Vassilis Stratoulias <vassilis.stratoulias@ki.se>; Kathleen Grabert <kathleen.grabert@ki.se>; Pinelopi Engskog Vlachos <pinelopi.vlachos@ki.se>; Jens Füllgrabe <jensfullgrabe@gmail.com>; Dan Klionsky <klionsky@umich.edu>
Ämne: Fw: CDDIS-22-3172RR Initial Quality Check

Dear Patricia, Elizabeth, Amelie, Lily, Vassilis, Kathleen, Poppi, Jens, and Dan,

Pinelopi Engskog-Vlachos in our laboratory who contributed to the generation of the new data set included during the revision of our manuscript entitled "SETD2 transcriptional control of ATG14L/S isoforms regulates autophagosome-lysosome fusion" to *Cell Death & Disease* has been added as co-author for her contribution.

The list of authors is as follows:
Patricia González-Rodríguez, Elizabeth Delorme-Axford, Amelie Bernard, Lily Keane, Vassilis Stratoulias, Kathleen Grabert, Pinelopi Engskog-Vlachos, Jens Füllgrabe, Daniel J. Klionsky, Bertrand Joseph.

Could you please all (including Poppi) confirm by return of email that you agree to this change in the list of authors? See request from the journal below.

I take the chance to thank you for our long-term successful and stimulating collaboration.

best regards

Bertrand

Bertrand Joseph | PhD
Professor of Molecular Cancer Biology

Institute of Environmental Medicine | Karolinska Institutet
Toxicology Unit, Nobelsväg 13
SE-171 77 Stockholm, Sweden
bertrand.joseph@ki.se | Joseph's Group | Karolinska Institutet

Karolinska Institutet is one of the world's leading medical universities. Its mission is to contribute to the improvement of human health through research and education. Karolinska Institutet accounts for over 40 per cent of the medical academic research conducted in Sweden and offers the country's broadest range of education in medicine and health sciences. Since 1901 the Nobel Assembly at Karolinska Institutet has selected the Nobel laureates in Physiology or Medicine.

From: cddisease@springernature.com <cddisease@springernature.com>
Sent: Monday, October 24, 2022 17:52
To: Bertrand Joseph <bertrand.joseph@ki.se>
Subject: CDDIS-22-3172RR Initial Quality Check

Dear Professor Joseph,

In checking in your manuscript submitted to Cell Death & Disease it has come to our attention that the following must be addressed before we can proceed.

1. It has come to our attention that your most recent author list differs from the one in your original submission. We find that the following authors have been added since your initial submission: Pinelopi Engskog-Vlachos

Please request agreement from all authors including additions and deletions, these can be collected in the following way:

Email your co-authors with the change, and ask them to reply to your email confirming that they agree to these changes. Once you have collected these replies, please combine all of the co-authors' email responses in one document and upload this file to your submission.

Your paper is now available for you to edit, you may access via the following link:

Please make the correction(s) as specified above and resubmit your paper following the same steps as before.

If you have any questions please feel free to contact us.

Sincerely,

Editorial Office
Cell Death & Disease
cddisease@springernature.com

This email has been sent through the NPG Manuscript Tracking System NY-610A-NPG&MTS

Confidentiality Statement:

This e-mail is confidential and subject to copyright. Any unauthorised use or disclosure of its contents is prohibited. If you have received this email in error please notify our Manuscript Tracking System Helpdesk team at http://platformsupport.nature.com .

Details of the confidentiality and pre-publicity policy may be found here http://www.nature.com/authors/policies/confidentiality.html

Privacy Policy | Update Profile

När du skickar e-post till Karolinska Institutet (KI) innebär detta att KI kommer att behandla dina personuppgifter. Här finns information om hur KI behandlar personuppgifter.

Sending email to Karolinska Institutet (KI) will result in KI processing your personal data. You can read more about KI's processing of personal data here.

Re: CDDIS-22-3172RR Initial Quality Check

Lily Keane <lily.keane@ki.se>
Tue 10/25/2022 10:35
To: Bertrand Joseph <bertrand.joseph@ki.se>;Pinelopi Engskog Vlachos <pinelopi.vlachos@ki.se>;Patricia Gonzalez-Rodriguez (UIO) <patricia.gonzalez-rodriguez@medisin.uio.no>;Elizabeth Delorme-Axford <delormeaxford@oakland.edu>;amelie.bernard@u-bordeaux.fr <amelie.bernard@u-bordeaux.fr>;Vassilis Stratoulias <vassilis.stratoulias@ki.se>;Kathleen Grabert <kathleen.grabert@ki.se>;Jens Füllgrabe <jensfullgrabe@gmail.com>;Dan Klionsky <klionsky@umich.edu>

Agree

Get Outlook for Android

From: Bertrand Joseph <bertrand.joseph@ki.se>
Sent: Tuesday, October 25, 2022 10:34:05 AM
To: Pinelopi Engskog Vlachos <pinelopi.vlachos@ki.se>; Patricia Gonzalez-Rodriguez (UIO) <patricia.gonzalez-rodriguez@medisin.uio.no>; Elizabeth Delorme-Axford <delormeaxford@oakland.edu>; amelie.bernard@u-bordeaux.fr <amelie.bernard@u-bordeaux.fr>; Lily Keane <lily.keane@ki.se>; Vassilis Stratoulias <vassilis.stratoulias@ki.se>; Kathleen Grabert <kathleen.grabert@ki.se>; Jens Füllgrabe <jensfullgrabe@gmail.com>; Dan Klionsky <klionsky@umich.edu>
Subject: Re: CDDIS-22-3172RR Initial Quality Check

Thank you!

From: Pinelopi Engskog Vlachos <pinelopi.vlachos@ki.se>
Sent: Tuesday, October 25, 2022 10:31
To: Bertrand Joseph <bertrand.joseph@ki.se>; Patricia Gonzalez-Rodriguez (UIO) <patricia.gonzalez-rodriguez@medisin.uio.no>; Elizabeth Delorme-Axford <delormeaxford@oakland.edu>; amelie.bernard@u-bordeaux.fr <amelie.bernard@u-bordeaux.fr>; Lily Keane <lily.keane@ki.se>; Vassilis Stratoulias <vassilis.stratoulias@ki.se>; Kathleen Grabert <kathleen.grabert@ki.se>; Jens Füllgrabe <jensfullgrabe@gmail.com>; Dan Klionsky <klionsky@umich.edu>
Subject: Sv: CDDIS-22-3172RR Initial Quality Check

Hi Bertrand
I agree to this change in the list of authors.

Best
Pinelopi Engskog Vlachos

Från: Bertrand Joseph <bertrand.joseph@ki.se>
Skickat: den 25 oktober 2022 10:23
Till: Patricia Gonzalez-Rodriguez (UIO) <patricia.gonzalez-rodriguez@medisin.uio.no>; Elizabeth Delorme-Axford <delormeaxford@oakland.edu>; amelie.bernard@u-bordeaux.fr; Lily Keane <lily.keane@ki.se>; Vassilis Stratoulias <vassilis.stratoulias@ki.se>; Kathleen Grabert <kathleen.grabert@ki.se>; Pinelopi Engskog Vlachos <pinelopi.vlachos@ki.se>; Jens Füllgrabe <jensfullgrabe@gmail.com>; Dan Klionsky <klionsky@umich.edu>
Ämne: Fw: CDDIS-22-3172RR Initial Quality Check

Dear Patricia, Elizabeth, Amelie, Lily, Vassilis, Kathleen, Poppi, Jens, and Dan,

Pinelopi Engskog-Vlachos in our laboratory who contributed to the generation of the new data set included during the revision of our manuscript entitled “SETD2 transcriptional control of ATG14L/S

isoforms regulates autophagosome-lysosome fusion" to *Cell Death & Disease* has been added as co-author for her contribution.

The list of authors is as follows:
Patricia González-Rodríguez, Elizabeth Delorme-Axford, Amelie Bernard, Lily Keane, Vassilis Stratoulias, Kathleen Grabert, Pinelopi Engskog-Vlachos, Jens Füllgrabe, Daniel J. Klionsky, Bertrand Joseph.

Could you please all (including Poppi) confirm by return of email that you agree to this change in the list of authors? See request from the journal below.

I take the chance to thank you for our long-term successful and stimulating collaboration.

best regards

Bertrand

Bertrand Joseph | PhD
Professor of Molecular Cancer Biology

Institute of Environmental Medicine | Karolinska Institutet
Toxicology Unit, Nobelsväg 13
SE-171 77 Stockholm, Sweden
bertrand.joseph@ki.se | Joseph's Group | Karolinska Institutet

---

Karolinska Institutet is one of the world's leading medical universities. Its mission is to contribute to the improvement of human health through research and education. Karolinska Institutet accounts for over 40 per cent of the medical academic research conducted in Sweden and offers the country's broadest range of education in medicine and health sciences. Since 1901 the Nobel Assembly at Karolinska Institutet has selected the Nobel laureates in Physiology or Medicine.

From: cddisease@springernature.com <cddisease@springernature.com>
Sent: Monday, October 24, 2022 17:52
To: Bertrand Joseph <bertrand.joseph@ki.se>
Subject: CDDIS-22-3172RR Initial Quality Check

Dear Professor Joseph,

In checking in your manuscript submitted to Cell Death & Disease it has come to our attention that the following must be addressed before we can proceed.

1. It has come to our attention that your most recent author list differs from the one in your original submission. We find that the following authors have been added since your initial submission: Pinelopi Engskog-Vlachos

Please request agreement from all authors including additions and deletions, these can be collected in the following way:

Email your co-authors with the change, and ask them to reply to your email confirming that they agree to these changes. Once you have collected these replies, please combine all of the co-authors' email responses in one document and upload this file to your submission.

Your paper is now available for you to edit, you may access via the following link:

Please make the correction(s) as specified above and resubmit your paper following the same steps as before.

If you have any questions please feel free to contact us.

Sincerely,

Editorial Office
Cell Death & Disease
cddisease@springernature.com

This email has been sent through the NPG Manuscript Tracking System NY-610A-NPG&MTS

Confidentiality Statement:

This e-mail is confidential and subject to copyright. Any unauthorised use or disclosure of its contents is prohibited. If you have received this email in error please notify our Manuscript Tracking System Helpdesk team at http://platformsupport.nature.com .
Details of the confidentiality and pre-publicity policy may be found here
http://www.nature.com/authors/policies/confidentiality.html
Privacy Policy | Update Profile

Delete Archive Report

RE: CDDIS-22-3172RR Initial Quality Check

Vassilis Stratoulias
To: Bertrand Joseph **+8 others**

Tue 10/25/2022 10:25

Agreed!

Thanks Pinelopi for contributing to this beautiful story ☺

BR

Vassilis

From: Bertrand Joseph <bertrand.joseph@ki.se>
Sent: 25 October 2022 11:23
To: Patricia Gonzalez-Rodriguez (UIO) <patricia.gonzalez-rodriguez@medisin.uio.no>; Elizabeth Delorme-Axford <delormeaxford@oakland.edu>; amelie.bernard@u-bordeaux.fr; Lily Keane <lily.keane@ki.se>; Vassilis Stratoulias <vassilis.stratoulias@ki.se>; Kathleen Grabert <kathleen.grabert@ki.se>; Pinelopi Engskog Vlachos <pinelopi.vlachos@ki.se>; Jens Füllgrabe <jensfullgrabe@gmail.com>; Dan Klionsky <klionsky@umich.edu>
Subject: Fw: CDDIS-22-3172RR Initial Quality Check

Dear Patricia, Elizabeth, Amelie, Lily, Vassilis, Kathleen, Poppi, Jens, and Dan,

Pinelopi Engskog-Vlachos in our laboratory who contributed to the generation of the new data set included during the revision of our manuscript entitled "SETD2 transcriptional control of ATG14L/S isoforms regulates autophagosome-lysosome fusion" to *Cell Death & Disease* has been added as co-author for her contribution.

The list of authors is as follows:
Patricia González-Rodríguez, Elizabeth Delorme-Axford, Amelie Bernard, Lily Keane, Vassilis Stratoulias, Kathleen Grabert, Pinelopi Engskog-Vlachos, Jens Füllgrabe, Daniel J. Klionsky, Bertrand Joseph.

Could you please all (including Poppi) confirm by return of email that you agree to this change in the list of authors? See request from the journal below.

I take the chance to thank you for our long-term successful and stimulating collaboration.

best regards

Bertrand

Bertrand Joseph | PhD
Professor of Molecular Cancer Biology

Institute of Environmental Medicine | Karolinska Institutet
Toxicology Unit, Nobelsväg 13

RE: CDDIS-22-3172RR Initial Quality Check

Kathleen Grabert <kathleen.grabert@ki.se>

Tue 10/25/2022 10:59

To: Lily Keane <lily.keane@ki.se>;Bertrand Joseph <bertrand.joseph@ki.se>;Pinelopi Engskog Vlachos <pinelopi.vlachos@ki.se>;Patricia Gonzalez-Rodriguez (UIO) <patricia.gonzalez-rodriguez@medisin.uio.no>;Elizabeth Delorme-Axford <delormeaxford@oakland.edu>;amelie.bernard@u-bordeaux.fr <amelie.bernard@u-bordeaux.fr>;Vassilis Stratoulias <vassilis.stratoulias@ki.se>;Jens Füllgrabe <jensfullgrabe@gmail.com>;Dan Klionsky <klionsky@umich.edu>

It's a YES from me!

From: Lily Keane <lily.keane@ki.se>
Sent: den 25 oktober 2022 10:36
To: Bertrand Joseph <bertrand.joseph@ki.se>; Pinelopi Engskog Vlachos <pinelopi.vlachos@ki.se>; Patricia Gonzalez-Rodriguez (UIO) <patricia.gonzalez-rodriguez@medisin.uio.no>; Elizabeth Delorme-Axford <delormeaxford@oakland.edu>; amelie.bernard@u-bordeaux.fr; Vassilis Stratoulias <vassilis.stratoulias@ki.se>; Kathleen Grabert <kathleen.grabert@ki.se>; Jens Füllgrabe <jensfullgrabe@gmail.com>; Dan Klionsky <klionsky@umich.edu>
Subject: Re: CDDIS-22-3172RR Initial Quality Check

Agree

Get Outlook for Android

From: Bertrand Joseph <bertrand.joseph@ki.se>
Sent: Tuesday, October 25, 2022 10:34:05 AM
To: Pinelopi Engskog Vlachos <pinelopi.vlachos@ki.se>; Patricia Gonzalez-Rodriguez (UIO) <patricia.gonzalez-rodriguez@medisin.uio.no>; Elizabeth Delorme-Axford <delormeaxford@oakland.edu>; amelie.bernard@u-bordeaux.fr <amelie.bernard@u-bordeaux.fr>; Lily Keane <lily.keane@ki.se>; Vassilis Stratoulias <vassilis.stratoulias@ki.se>; Kathleen Grabert <kathleen.grabert@ki.se>; Jens Füllgrabe <jensfullgrabe@gmail.com>; Dan Klionsky <klionsky@umich.edu>
Subject: Re: CDDIS-22-3172RR Initial Quality Check

Thank you!

From: Pinelopi Engskog Vlachos <pinelopi.vlachos@ki.se>
Sent: Tuesday, October 25, 2022 10:31
To: Bertrand Joseph <bertrand.joseph@ki.se>; Patricia Gonzalez-Rodriguez (UIO) <patricia.gonzalez-rodriguez@medisin.uio.no>; Elizabeth Delorme-Axford <delormeaxford@oakland.edu>; amelie.bernard@u-bordeaux.fr <amelie.bernard@u-bordeaux.fr>; Lily Keane <lily.keane@ki.se>; Vassilis Stratoulias <vassilis.stratoulias@ki.se>; Kathleen Grabert <kathleen.grabert@ki.se>; Jens Füllgrabe <jensfullgrabe@gmail.com>; Dan Klionsky <klionsky@umich.edu>
Subject: Sv: CDDIS-22-3172RR Initial Quality Check

Hi Bertrand
I agree to this change in the list of authors.

Best
Pinelopi Engskog Vlachos

Från: Bertrand Joseph <bertrand.joseph@ki.se>
Skickat: den 25 oktober 2022 10:23
Till: Patricia Gonzalez-Rodriguez (UIO) <patricia.gonzalez-rodriguez@medisin.uio.no>; Elizabeth Delorme-

Axford <delormeaxford@oakland.edu>; amelie.bernard@u-bordeaux.fr; Lily Keane <lily.keane@ki.se>; Vassilis Stratoulias <vassilis.stratoulias@ki.se>; Kathleen Grabert <kathleen.grabert@ki.se>; Pinelopi Engskog Vlachos <pinelopi.vlachos@ki.se>; Jens Füllgrabe <jensfullgrabe@gmail.com>; Dan Klionsky <klionsky@umich.edu>
Ämne: Fw: CDDIS-22-3172RR Initial Quality Check

Dear Patricia, Elizabeth, Amelie, Lily, Vassilis, Kathleen, Poppi, Jens, and Dan,

Pinelopi Engskog-Vlachos in our laboratory who contributed to the generation of the new data set included during the revision of our manuscript entitled "SETD2 transcriptional control of ATG14L/S isoforms regulates autophagosome-lysosome fusion" to *Cell Death & Disease* has been added as co-author for her contribution.

The list of authors is as follows:
Patricia González-Rodríguez, Elizabeth Delorme-Axford, Amelie Bernard, Lily Keane, Vassilis Stratoulias, Kathleen Grabert, Pinelopi Engskog-Vlachos, Jens Füllgrabe, Daniel J. Klionsky, Bertrand Joseph.

Could you please all (including Poppi) confirm by return of email that you agree to this change in the list of authors? See request from the journal below.

I take the chance to thank you for our long-term successful and stimulating collaboration.

best regards

Bertrand

Bertrand Joseph | PhD
Professor of Molecular Cancer Biology

Institute of Environmental Medicine | Karolinska Institutet
Toxicology Unit, Nobelsväg 13
SE-171 77 Stockholm, Sweden
bertrand.joseph@ki.se | Joseph's Group | Karolinska Institutet

---

Karolinska Institutet is one of the world's leading medical universities. Its mission is to contribute to the improvement of human health through research and education. Karolinska Institutet accounts for over 40 per cent of the medical academic research conducted in Sweden and offers the country's broadest range of education in medicine and health sciences. Since 1901 the Nobel Assembly at Karolinska Institutet has selected the Nobel laureates in Physiology or Medicine.

From: cddisease@springernature.com <cddisease@springernature.com>
Sent: Monday, October 24, 2022 17:52
To: Bertrand Joseph <bertrand.joseph@ki.se>
Subject: CDDIS-22-3172RR Initial Quality Check

Dear Professor Joseph,

In checking in your manuscript submitted to Cell Death & Disease it has come to our attention that the following must be addressed before we can proceed.

1. It has come to our attention that your most recent author list differs from the one in your original submission. We find that the following authors have been added since your initial submission: Pinelopi

Engskog-Vlachos

Please request agreement from all authors including additions and deletions, these can be collected in the following way:

Email your co-authors with the change, and ask them to reply to your email confirming that they agree to these changes. Once you have collected these replies, please combine all of the co-authors' email responses in one document and upload this file to your submission.

Your paper is now available for you to edit, you may access via the following link:

Please make the correction(s) as specified above and resubmit your paper following the same steps as before.

If you have any questions please feel free to contact us.

Sincerely,

Editorial Office
Cell Death & Disease
cddisease@springernature.com

This email has been sent through the NPG Manuscript Tracking System NY-610A-NPG&MTS

Confidentiality Statement:

This e-mail is confidential and subject to copyright. Any unauthorised use or disclosure of its contents is prohibited. If you have received this email in error please notify our Manuscript Tracking System Helpdesk team at http://platformsupport.nature.com .

Details of the confidentiality and pre-publicity policy may be found here http://www.nature.com/authors/policies/confidentiality.html

Privacy Policy | Update Profile

Sv: CDDIS-22-3172RR Initial Quality Check

Pinelopi Engskog Vlachos <pinelopi.vlachos@ki.se>
Tue 10/25/2022 10:31

To: Bertrand Joseph <bertrand.joseph@ki.se>;Patricia Gonzalez-Rodriguez (UIO) <patricia.gonzalez-rodriguez@medisin.uio.no>;Elizabeth Delorme-Axford <delormeaxford@oakland.edu>;amelie.bernard@u-bordeaux.fr <amelie.bernard@u-bordeaux.fr>;Lily Keane <lily.keane@ki.se>;Vassilis Stratoulias <vassilis.stratoulias@ki.se>;Kathleen Grabert <kathleen.grabert@ki.se>;Jens Füllgrabe <jensfullgrabe@gmail.com>;Dan Klionsky <klionsky@umich.edu>

Hi Bertrand
I agree to this change in the list of authors.

Best
Pinelopi Engskog Vlachos

Från: Bertrand Joseph <bertrand.joseph@ki.se>
Skickat: den 25 oktober 2022 10:23
Till: Patricia Gonzalez-Rodriguez (UIO) <patricia.gonzalez-rodriguez@medisin.uio.no>; Elizabeth Delorme-Axford <delormeaxford@oakland.edu>; amelie.bernard@u-bordeaux.fr; Lily Keane <lily.keane@ki.se>; Vassilis Stratoulias <vassilis.stratoulias@ki.se>; Kathleen Grabert <kathleen.grabert@ki.se>; Pinelopi Engskog Vlachos <pinelopi.vlachos@ki.se>; Jens Füllgrabe <jensfullgrabe@gmail.com>; Dan Klionsky <klionsky@umich.edu>
Ämne: Fw: CDDIS-22-3172RR Initial Quality Check

Dear Patricia, Elizabeth, Amelie, Lily, Vassilis, Kathleen, Poppi, Jens, and Dan,

Pinelopi Engskog-Vlachos in our laboratory who contributed to the generation of the new data set included during the revision of our manuscript entitled "SETD2 transcriptional control of ATG14L/S isoforms regulates autophagosome-lysosome fusion" to *Cell Death & Disease* has been added as co-author for her contribution.

The list of authors is as follows:
Patricia González-Rodríguez, Elizabeth Delorme-Axford, Amelie Bernard, Lily Keane, Vassilis Stratoulias, Kathleen Grabert, Pinelopi Engskog-Vlachos, Jens Füllgrabe, Daniel J. Klionsky, Bertrand Joseph.

Could you please all (including Poppi) confirm by return of email that you agree to this change in the list of authors? See request from the journal below.

I take the chance to thank you for our long-term successful and stimulating collaboration.

best regards

Bertrand

Bertrand Joseph | PhD
Professor of Molecular Cancer Biology

Institute of Environmental Medicine | Karolinska Institutet
Toxicology Unit, Nobelsväg 13

SE-171 77 Stockholm, Sweden
bertrand.joseph@ki.se | Joseph's Group | Karolinska Institutet

---

Karolinska Institutet is one of the world's leading medical universities. Its mission is to contribute to the improvement of human health through research and education. Karolinska Institutet accounts for over 40 per cent of the medical academic research conducted in Sweden and offers the country's broadest range of education in medicine and health sciences. Since 1901 the Nobel Assembly at Karolinska Institutet has selected the Nobel laureates in Physiology or Medicine.

From: cddisease@springernature.com <cddisease@springernature.com>
Sent: Monday, October 24, 2022 17:52
To: Bertrand Joseph <bertrand.joseph@ki.se>
Subject: CDDIS-22-3172RR Initial Quality Check

Dear Professor Joseph,

In checking in your manuscript submitted to Cell Death & Disease it has come to our attention that the following must be addressed before we can proceed.

1. It has come to our attention that your most recent author list differs from the one in your original submission. We find that the following authors have been added since your initial submission: Pinelopi Engskog-Vlachos

Please request agreement from all authors including additions and deletions, these can be collected in the following way:

Email your co-authors with the change, and ask them to reply to your email confirming that they agree to these changes. Once you have collected these replies, please combine all of the co-authors' email responses in one document and upload this file to your submission.

Your paper is now available for you to edit, you may access via the following link:

Please make the correction(s) as specified above and resubmit your paper following the same steps as before.

If you have any questions please feel free to contact us.

Sincerely,

Editorial Office
Cell Death & Disease
cddisease@springernature.com

This email has been sent through the NPG Manuscript Tracking System NY-610A-NPG&MTS

Confidentiality Statement:

This e-mail is confidential and subject to copyright. Any unauthorised use or disclosure of its contents is prohibited. If you have received this email in error please notify our Manuscript Tracking System Helpdesk team at http://platformsupport.nature.com .

Details of the confidentiality and pre-publicity policy may be found here
http://www.nature.com/authors/policies/confidentiality.html

Privacy Policy | Update Profile

Re: CDDIS-22-3172RR Initial Quality Check

Jens Füllgrabe <jensfullgrabe@gmail.com>
Tue 10/25/2022 13:27
To: Bertrand Joseph <bertrand.joseph@ki.se>
Cc: Kathleen Grabert <kathleen.grabert@ki.se>;Lily Keane <lily.keane@ki.se>;Pinelopi Engskog Vlachos <pinelopi.vlachos@ki.se>;Patricia Gonzalez-Rodriguez (UIO) <patricia.gonzalez-rodriguez@medisin.uio.no>;Elizabeth Delorme-Axford <delormeaxford@oakland.edu>;amelie.bernard@u-bordeaux.fr <amelie.bernard@u-bordeaux.fr>;Vassilis Stratoulias <vassilis.stratoulias@ki.se>;Dan Klionsky <klionsky@umich.edu>

Hi,

Yes, of course.

Kind regards,
Jens

On Tue, 25 Oct 2022, 11:29 Bertrand Joseph, <bertrand.joseph@ki.se> wrote:

> Great, thanks!
>
> ---
>
> **From:** Kathleen Grabert <kathleen.grabert@ki.se>
> **Sent:** Tuesday, October 25, 2022 10:59
> **To:** Lily Keane <lily.keane@ki.se>; Bertrand Joseph <bertrand.joseph@ki.se>; Pinelopi Engskog Vlachos <pinelopi.vlachos@ki.se>; Patricia Gonzalez-Rodriguez (UIO) <patricia.gonzalez-rodriguez@medisin.uio.no>; Elizabeth Delorme-Axford <delormeaxford@oakland.edu>; amelie.bernard@u-bordeaux.fr <amelie.bernard@u-bordeaux.fr>; Vassilis Stratoulias <vassilis.stratoulias@ki.se>; Jens Füllgrabe <jensfullgrabe@gmail.com>; Dan Klionsky <klionsky@umich.edu>
> **Subject:** RE: CDDIS-22-3172RR Initial Quality Check
>
> It's a YES from me!
>
> ---
>
> **From:** Lily Keane <lily.keane@ki.se>
> **Sent:** den 25 oktober 2022 10:36
> **To:** Bertrand Joseph <bertrand.joseph@ki.se>; Pinelopi Engskog Vlachos <pinelopi.vlachos@ki.se>; Patricia Gonzalez-Rodriguez (UIO) <patricia.gonzalez-rodriguez@medisin.uio.no>; Elizabeth Delorme-Axford <delormeaxford@oakland.edu>; amelie.bernard@u-bordeaux.fr; Vassilis Stratoulias <vassilis.stratoulias@ki.se>; Kathleen Grabert <kathleen.grabert@ki.se>; Jens Füllgrabe <jensfullgrabe@gmail.com>; Dan Klionsky <klionsky@umich.edu>
> **Subject:** Re: CDDIS-22-3172RR Initial Quality Check
>
> Agree

Get Outlook for Android

From: Bertrand Joseph <bertrand.joseph@ki.se>
Sent: Tuesday, October 25, 2022 10:34:05 AM
To: Pinelopi Engskog Vlachos <pinelopi.vlachos@ki.se>; Patricia Gonzalez-Rodriguez (UIO) <patricia.gonzalez-rodriguez@medisin.uio.no>; Elizabeth Delorme-Axford <delormeaxford@oakland.edu>; amelie.bernard@u-bordeaux.fr <amelie.bernard@u-bordeaux.fr>; Lily Keane <lily.keane@ki.se>; Vassilis Stratoulias <vassilis.stratoulias@ki.se>; Kathleen Grabert <kathleen.grabert@ki.se>; Jens Füllgrabe <jensfullgrabe@gmail.com>; Dan Klionsky <klionsky@umich.edu>
Subject: Re: CDDIS-22-3172RR Initial Quality Check

Thank you!

From: Pinelopi Engskog Vlachos <pinelopi.vlachos@ki.se>
Sent: Tuesday, October 25, 2022 10:31
To: Bertrand Joseph <bertrand.joseph@ki.se>; Patricia Gonzalez-Rodriguez (UIO) <patricia.gonzalez-rodriguez@medisin.uio.no>; Elizabeth Delorme-Axford <delormeaxford@oakland.edu>; amelie.bernard@u-bordeaux.fr <amelie.bernard@u-bordeaux.fr>; Lily Keane <lily.keane@ki.se>; Vassilis Stratoulias <vassilis.stratoulias@ki.se>; Kathleen Grabert <kathleen.grabert@ki.se>; Jens Füllgrabe <jensfullgrabe@gmail.com>; Dan Klionsky <klionsky@umich.edu>
Subject: Sv: CDDIS-22-3172RR Initial Quality Check

Hi Bertrand

I agree to this change in the list of authors.

Best

Pinelopi Engskog Vlachos

Från: Bertrand Joseph <bertrand.joseph@ki.se>
Skickat: den 25 oktober 2022 10:23
Till: Patricia Gonzalez-Rodriguez (UIO) <patricia.gonzalez-rodriguez@medisin.uio.no>; Elizabeth Delorme-Axford <delormeaxford@oakland.edu>; amelie.bernard@u-bordeaux.fr; Lily Keane <lily.keane@ki.se>; Vassilis Stratoulias <vassilis.stratoulias@ki.se>; Kathleen Grabert <kathleen.grabert@ki.se>; Pinelopi Engskog Vlachos <pinelopi.vlachos@ki.se>; Jens Füllgrabe <jensfullgrabe@gmail.com>; Dan Klionsky <klionsky@umich.edu>
Ämne: Fw: CDDIS-22-3172RR Initial Quality Check

Dear Patricia, Elizabeth, Amelie, Lily, Vassilis, Kathleen, Poppi, Jens, and Dan,

Pinelopi Engskog-Vlachos in our laboratory who contributed to the generation of the new data set included during the revision of our manuscript entitled "SETD2 transcriptional control of ATG14L/S isoforms regulates autophagosome-lysosome fusion" to *Cell Death & Disease* has been added as co-author for her contribution.

The list of authors is as follows:

Patricia González-Rodríguez, Elizabeth Delorme-Axford, Amelie Bernard, Lily Keane, Vassilis Stratoulias, Kathleen Grabert, Pinelopi Engskog-Vlachos, Jens Füllgrabe, Daniel J. Klionsky, Bertrand Joseph.

Could you please all (including Poppi) confirm by return of email that you agree to this change in the list of authors? See request from the journal below.

I take the chance to thank you for our long-term successful and stimulating collaboration.

best regards

Bertrand

Bertrand Joseph | PhD
Professor of Molecular Cancer Biology

Institute of Environmental Medicine | Karolinska Institutet
Toxicology Unit, Nobelsväg 13
SE-171 77 Stockholm, Sweden
bertrand.joseph@ki.se | Joseph's Group | Karolinska Institutet

---

Karolinska Institutet is one of the world's leading medical universities. Its mission is to contribute to the improvement of human health through research and education. Karolinska Institutet accounts for over 40 per cent of the medical academic research conducted in Sweden and offers the country's broadest range of education in medicine and health sciences. Since 1901 the Nobel Assembly at Karolinska Institutet has selected the Nobel laureates in Physiology or Medicine.

From: cddisease@springernature.com <cddisease@springernature.com>
Sent: Monday, October 24, 2022 17:52
To: Bertrand Joseph <bertrand.joseph@ki.se>
Subject: CDDIS-22-3172RR Initial Quality Check

Dear Professor Joseph,

In checking in your manuscript submitted to Cell Death & Disease it has come to our attention that the following must be addressed before we can proceed.

1. It has come to our attention that your most recent author list differs from the one in your original submission. We find that the following authors have been added since your initial submission: Pinelopi Engskog-Vlachos

Please request agreement from all authors including additions and deletions, these can be collected in the following way:

Email your co-authors with the change, and ask them to reply to your email confirming that they agree to these changes. Once you have collected these replies, please combine all of the co-authors' email responses in one document and upload this file to your submission.

Your paper is now available for you to edit, you may access via the following link:

Please make the correction(s) as specified above and resubmit your paper following the same steps as before.

If you have any questions please feel free to contact us.

Sincerely,

Editorial Office
Cell Death & Disease
cddisease@springernature.com

This email has been sent through the NPG Manuscript Tracking System NY-610A-NPG&MTS

Confidentiality Statement:

This e-mail is confidential and subject to copyright. Any unauthorised use or disclosure of its contents is prohibited. If you have received this email in error please notify our Manuscript Tracking System Helpdesk team at http://platformsupport.nature.com .

Details of the confidentiality and pre-publicity policy may be found here http://www.nature.com/authors/policies/confidentiality.html

Privacy Policy | Update Profile

När du skickar e-post till Karolinska Institutet (KI) innebär detta att KI kommer att behandla dina personuppgifter. Här finns information om hur KI behandlar personuppgifter.

Sending email to Karolinska Institutet (KI) will result in KI processing your personal data. You can read more about KI's processing of personal data here.

Re: Fw: CDDIS-22-3172RR Initial Quality Check

Daniel Klionsky <klionsky@umich.edu>
Tue 10/25/2022 12:48
To: Bertrand Joseph <bertrand.joseph@ki.se>
Cc: Patricia Gonzalez-Rodriguez (UIO) <patricia.gonzalez-rodriguez@medisin.uio.no>;Elizabeth Delorme-Axford <delormeaxford@oakland.edu>;amelie.bernard@u-bordeaux.fr <amelie.bernard@u-bordeaux.fr>;Lily Keane <lily.keane@ki.se>;Vassilis Stratoulias <vassilis.stratoulias@ki.se>;Kathleen Grabert <kathleen.grabert@ki.se>;Pinelopi Engskog Vlachos <pinelopi.vlachos@ki.se>;Jens Füllgrabe <jensfullgrabe@gmail.com>

Yes, I agree.

Dan

On Tue, Oct 25, 2022 at 4:23 AM Bertrand Joseph <bertrand.joseph@ki.se> wrote:

Dear Patricia, Elizabeth, Amelie, Lily, Vassilis, Kathleen, Poppi, Jens, and Dan,

Pinelopi Engskog-Vlachos in our laboratory who contributed to the generation of the new data set included during the revision of our manuscript entitled "SETD2 transcriptional control of ATG14L/S isoforms regulates autophagosome-lysosome fusion" to *Cell Death & Disease* has been added as co-author for her contribution.

The list of authors is as follows:
Patricia González-Rodríguez, Elizabeth Delorme-Axford, Amelie Bernard, Lily Keane, Vassilis Stratoulias, Kathleen Grabert, Pinelopi Engskog-Vlachos, Jens Füllgrabe, Daniel J. Klionsky, Bertrand Joseph.

Could you please all (including Poppi) confirm by return of email that you agree to this change in the list of authors? See request from the journal below.

I take the chance to thank you for our long-term successful and stimulating collaboration.

best regards

Bertrand

Bertrand Joseph | PhD
Professor of Molecular Cancer Biology

Institute of Environmental Medicine | Karolinska Institutet
Toxicology Unit, Nobelsväg 13
SE-171 77 Stockholm, Sweden
bertrand.joseph@ki.se | Joseph's Group | Karolinska Institutet

Karolinska Institutet is one of the world's leading medical universities. Its mission is to contribute to the improvement of human health through research and education. Karolinska Institutet accounts for over 40 per cent of the medical academic research conducted in Sweden and offers the country's broadest range of education in medicine and health sciences. Since 1901 the Nobel Assembly at Karolinska Institutet has selected the Nobel laureates in Physiology or Medicine.

From: cddisease@springernature.com <cddisease@springernature.com>
Sent: Monday, October 24, 2022 17:52
To: Bertrand Joseph <bertrand.joseph@ki.se>
Subject: CDDIS-22-3172RR Initial Quality Check

Dear Professor Joseph,

In checking in your manuscript submitted to Cell Death & Disease it has come to our attention that the following must be addressed before we can proceed.

1. It has come to our attention that your most recent author list differs from the one in your original submission. We find that the following authors have been added since your initial submission: Pinelopi Engskog-Vlachos

Please request agreement from all authors including additions and deletions, these can be collected in the following way:

Email your co-authors with the change, and ask them to reply to your email confirming that they agree to these changes. Once you have collected these replies, please combine all of the co-authors' email responses in one document and upload this file to your submission.

Your paper is now available for you to edit, you may access via the following link:

Please make the correction(s) as specified above and resubmit your paper following the same steps as before.

If you have any questions please feel free to contact us.

Sincerely,

Editorial Office
Cell Death & Disease
cddisease@springernature.com

This email has been sent through the NPG Manuscript Tracking System NY-610A-NPG&MTS

Confidentiality Statement:

This e-mail is confidential and subject to copyright. Any unauthorised use or disclosure of its contents is prohibited. If you have received this email in error please notify our Manuscript Tracking System Helpdesk team at http://platformsupport.nature.com .

Details of the confidentiality and pre-publicity policy may be found here http://www.nature.com/authors/policies/confidentiality.html

Privacy Policy | Update Profile

När du skickar e-post till Karolinska Institutet (KI) innebär detta att KI kommer att behandla dina personuppgifter. Här finns information om hur KI behandlar personuppgifter.

Sending email to Karolinska Institutet (KI) will result in KI processing your personal data. You can read more about KI's processing of personal data here.
